# Elucidating the Role of O_2_ Uncoupling for the Adaptation of Bacterial Biodegradation Reactions Catalyzed by Rieske Oxygenases

**DOI:** 10.1021/acsenvironau.4c00016

**Published:** 2024-05-14

**Authors:** Charlotte E. Bopp, Nora M. Bernet, Fabian Meyer, Riyaz Khan, Serina L. Robinson, Hans-Peter E. Kohler, Rebecca Buller, Thomas B. Hofstetter

**Affiliations:** † Eawag, Swiss Federal Institute of Aquatic Science and Technology, 8600 Dübendorf, Switzerland; ‡ Institute of Biogeochemistry and Pollutant Dynamics (IBP), ETH Zürich, 8092 Zürich, Switzerland; § Competence Center for Biocatalysis, Institute of Chemistry and Biotechnology, Zürich University of Applied Sciences, 8820 Wädenswil, Switzerland

**Keywords:** Rieske non-heme ferrous iron oxygenases, biocatalysis, O_2_ activation and uncoupling, reactive oxygen species, biodegradation, evolutionary adaptation

## Abstract

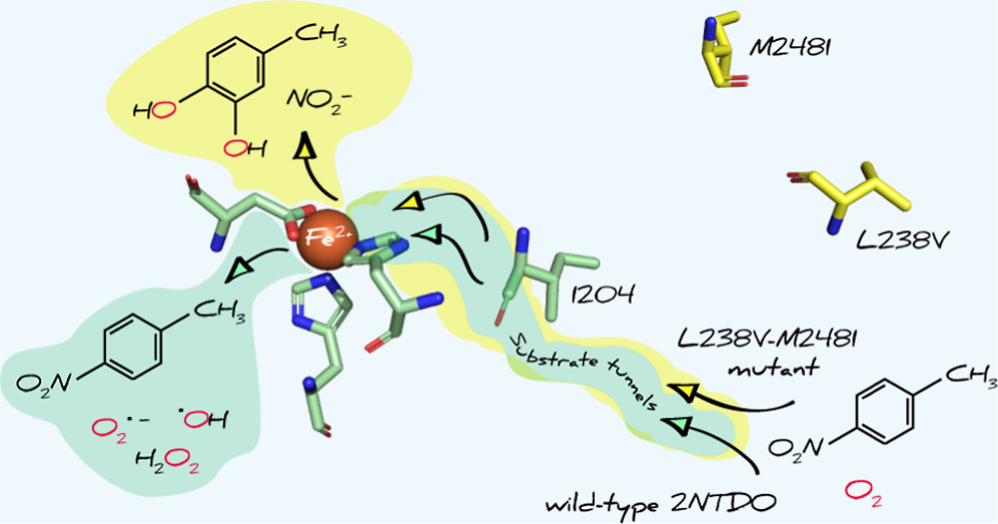

Oxygenation of aromatic and aliphatic hydrocarbons by Rieske oxygenases is the initial step of various biodegradation pathways for environmental organic contaminants. Microorganisms carrying Rieske oxygenases are able to quickly adapt their substrate spectra to alternative carbon and energy sources that are structurally related to the original target substrate, yet the molecular events responsible for this rapid adaptation are not well understood. Here, we evaluated the hypothesis that reactive oxygen species (ROS) generated by unproductive activation of O_2_, the so-called O_2_ uncoupling, in the presence of the alternative substrate exert a selective pressure on the bacterium for increasing the oxygenation efficiency of Rieske oxygenases. To that end, we studied wild-type 2-nitrotoluene dioxygenase from *Acidovorax* sp. strain JS42 and five enzyme variants that have evolved from adaptive laboratory evolution experiments with 3- and 4-nitrotoluene as alternative growth substrates. The enzyme variants showed a substantially increased oxygenation efficiency toward the new target substrates concomitant with a reduction of ROS production, while mechanisms and kinetics of enzymatic O_2_ activation remained unchanged. Structural analyses and docking studies suggest that amino acid substitutions in enzyme variants occurred at residues lining both substrate and O_2_ transport tunnels, enabling tighter binding of the target substrates in the active site. Increased oxygenation efficiencies measured in vitro for the various enzyme (variant)-substrate combinations correlated linearly with in vivo changes in growth rates for evolved *Acidovorax* strains expressing the variants. Our data suggest that the selective pressure from oxidative stress toward more efficient oxygenation by Rieske oxygenases was most notable when O_2_ uncoupling exceeded 60%.

## Introduction

Understanding biodegradation processes of anthropogenic organic contaminants in natural and engineered environments including soils, sediments, as well as (waste)water treatment plants is a critical step for designing measures that maintain ecosystem services and sustainable access to food and water under increasing human impact.^1−5^ Contaminant biodegradation routes often involve redox reactions with molecular O_2_ as the oxidant. These enzymatic oxygenations of anthropogenic organic contaminants lead to oxygenated products that feed into standard catabolic and biosynthetic pathways.^6−9^ Genomic information suggests that the ability of microbes to transform such contaminants, that is, the biodegradation potential, could even be larger than hypothesized on the basis of known enzymes and their function.^10−13^ However, predicting the occurrence of oxidative biodegradation based on genomic inferences of oxygenase abundances in the environment is particularly difficult. On the one hand, the sequence and kinetics of elementary steps leading to O_2_ activation and substrate hydroxylation are often enzyme-specific^14−22^ and therefore preclude generalized predictions of pathways, rates, and extents of biodegradation.^23−25^ On the other hand, the substrate specificity is highly variable, even for structurally related enzymes,^24−28^ and subject to evolutionary pressure. Environmental microbiomes are continuously exposed to a broad variety of organic contaminants that cause microbes to adapt their metabolism to xenobiotic compounds as novel substrates and metagenomic information indeed hints at ongoing sequence mutations.^10,11,29−36^

It is hypothesized that the evolution of oxygenases that can transform new contaminants in biodegradation processes is driven by the erroneous handling of activated oxygen and the ensuing release of reactive oxygen species (ROS),^37−39^ a phenomenon referred to as O_2_ uncoupling.^40−45^ Reactions with ROS can, in fact, inactivate proteins. Different ROS-scavenging enzymes and repair systems are therefore in place to mitigate the general oxidative stress associated with metabolic reactions involving O_2_.^46−49^ However, it is so far unclear whether alterations of protein sequences and structures emerge as a consequence of ROS formation in O_2_-activating enzymes and whether this process ultimately results in a reduced O_2_ uncoupling as microorganisms adapt to new substrates. For protecting themselves against oxidative damage from ROS, many oxidoreductases can transport potentially damaging oxidizing equivalents away from active sites toward the protein surface through redox-active tyrosine/tryptophan chains.^50−54^ Consistent with this hole hopping process and the notion that cellular reductants would scavenge these oxidation equivalents, reconfiguration of metabolic fluxes has been observed upon ROS release from the oxygenase concomitant with faster turnover of reduction equivalents.^55^ Thus, even in the presence of defense mechanisms at the enzyme level, modifications of contaminant-degrading oxygenases that allow for reduced extents of ROS formation would appear (at least) metabolically beneficial to the oxygenase-expressing microorganism. These oxygenases enable access to an additional, potentially broad substrate spectrum of environmental contaminants. Several contaminant-degrading oxygenases are considered promiscuous^56−59^ and their alteration to accept alternative substrates constitutes a competitive advantage for the respective microorganisms.^11^ However, it is not known whether a modification of contaminant-degrading oxygenases as a consequence of exposure to xenobiotic substrates is indeed accompanied by reduced O_2_ uncoupling and concomitant decrease of ROS formation.

In this study, we aimed to establish the relationship between microbial adaptation to alternative substrates, modification of enzyme sequence and structure, and the oxygenation efficiency of contaminant-degrading oxygenases. To that end, we studied Rieske non-heme ferrous iron oxygenases,^20,60−65^ an important class of contaminant-degrading enzymes. Rieske oxygenases catalyze oxygenations and oxidative heteroatom dealkylations of a broad number of contaminants including carboxylated, nitrated, halogenated, as well as *N*- and *O*-alkylated (poly)aromatic structures.^64−79^ These substrate structures reflect the wide range of man-made chemicals such as pesticides, pharmaceuticals, industrial chemicals, and explosives.^40,56,58,80−86^ Despite the well-known role of Rieske oxygenases in biocatalysis, the factors that lead to successful substrate oxygenation are still largely elusive. Both structural elements of the oxygenase such as substrate tunnels and flexible loops as well as electronic interactions in the active site between the substrate and the non-heme Fe center appear critical for successful O_2_ activation and substrate hydroxylation.^64,66,70,82,87−94^ Yet, it remains unclear whether any of these structural and electronic factors are optimized in Rieske oxygenases as microorganisms adapt to alternative contaminants as primary substrates.

Previous laboratory evolution experiments with nitroarene dioxygenases^95−99^ offer a promising avenue to examine the above questions. These works showed that *Acidovorax* sp. strain JS42 expressing the Rieske oxygenase 2-nitrotoluene dioxygenase (2NTDO) altered its substrate specificity from 2-nitrotoluene to 3- and 4-nitrotoluene as growth-supporting substrates if exposed to one of these alternative substrates over weeks and months, respectively. This adaptation process was accompanied by one and two amino acid substitutions in the enzyme variants compared to the wild-type (wt) 2NTDO. Note, however, that selective sequencing of the *ntdAcAd* genes encoding this oxygenase precluded the identification of additional mutations related to ROS defense mechanisms. We recently quantified in vitro oxygenation efficiencies of Rieske oxygenases as the fraction of consumed O_2_ used for substrate hydroxylation. Our studies of oxygenation efficiencies of wt 2NTDO revealed that the share of productively activated O_2_ by 2NTDO with 3- and 4-nitrotoluene amounts to less than 16 and 6%, respectively. Conversely, 98% of O_2_ was recovered in hydroxylation products when 2-nitrotoluene was used as the substrate.^24^

Here, we revisit the work of Parales et al.^95,96^ to examine two hypotheses regarding the adaptation of 2NTDO toward oxygenation of 3- and 4-nitrotoluene. (i) Enzyme evolution in such biodegradation processes correlates with increasingly efficient substrate hydroxylation and thus reduction of ROS generation. (ii) This process occurs as substitutions at residues that are located in structural “hotspots” for catalytic performance.^64,70,91,100^ To that end, we carried out the following three tasks. (1) We quantified the substrate oxygenation efficiencies for four nitroaromatic substrates with wt 2NTDO as well as five adapted variants exhibiting single and double amino acid substitutions^95,96^ and studied their relevance for enabling growth of *Acidovorax* sp. strain JS42. (2) We examined whether the kinetic mechanism associated with the catalytic cycle of Rieske oxygenases^25,101,102^ was conserved in enzyme variants by evaluating the kinetics of O_2_ activation and substrate hydroxylation. (3) We analyzed whether the amino acid substitutions are localized at or near the active site or in substrate transport tunnels and whether these structural modifications modulate substrate binding and/or intramolecular substrate transport^103,104^ in ways that could affect the extent of O_2_ uncoupling. Finally, we assessed our results in an environmental context by examining the relevance of the variants identified by Parales et al.^95,96^ through the bioinformatic analysis of naturally occurring Rieske oxygenase sequences.

## Experimental and Computational Methods

All chemicals and materials used are reported in Section S1 of the Supporting Information. Enzyme purification, experimental, and analytical procedures are identical to methods described before by Bopp et al.^24^

### Bacterial Strains and Site-Directed Mutagenesis

*Escherichia coli* DH5α expressing recombinant dioxygenases from plasmids pKSJ90 (2NTDO M248I) and pKSJ92 (2NTDO L238V-M248I) were obtained from Rebecca E. Parales.^95^ Plasmids corresponding to pKMM32, pKMM33, and pKMM35^96^ carrying the *ntdAcAd* genes for the I204A, I204T, and I204V variants of 2NTDO, respectively, were obtained from site-directed mutagenesis using pDTG800^99^ carrying the *ntdAcAd* genes of wt 2NTDO as template. We performed site-directed mutagenesis using KLD mix (New England BioLabs) with forward primers including the exchanged codon and nonoverlapping reverse primers that are listed in Table S1. Incubation and purification procedures followed the methods described for NBDO and 2NTDO.^24^ Activities, purities, and yields were comparable to those for 2NTDO. A comparison of the two amino acid sequences of the catalytically active α subunits of NBDO and 2NTDO is shown in Figure S1.

### Enzyme Assays

NADH-limited enzyme assays were used to determine the turnover of nitroaromatic substrates to organic and inorganic reaction products, O_2_ disappearance, and H_2_O_2_ formation, as well as changes in ^13^C/^12^C ratios of the organic substrate and ^18^O/^16^O ratios of dissolved O_2_. Experiments were carried out in 12 mL crimp-top vials filled completely with 50 mM MES buffer (pH 6.8), 0.15 μM reductase, 1.8 μM ferredoxin, 0.15 μM oxygenase, 100 μM (NH_4_)_2_Fe(SO_4_)_2_, and 90–200 μM of nitroaromatic substrate at initial dissolved O_2_ concentrations of 260–280 μM. Under constant monitoring of O_2_ concentrations, reactions were initiated by the addition of 10–50 μL of 50 mM NADH stock (in 10 mM NaOH) and run until dissolved O_2_ reached constant concentrations. Each set of experiments consisted of four to six separate enzyme assays with different initial NADH concentrations (40–250 μM) and two blanks without NADH for experimental determination of the initial concentration of the substrates. From selected enzyme-substrate combinations, we also quantified H_2_O_2_ formation using a horse radish peroxidase (HRP) assay to catalyze the reduction of H_2_O_2_ by oxidizing 4-methoxyaniline (Section S2.4).^24,25^ 900 μL was withdrawn from each NADH-limited enzyme assay and mixed with 100 μL of an HRP assay in MES buffer to final concentrations of 10 mg L^–1^ HRP and 500 μM 4-methoxyaniline. Kinetics of O_2_ consumption and substrate oxygenation were performed in modified assays as described before^24,25^ and in Supporting Information Sections S2.2 and S2.3.

### Chemical and Isotopic Analyses

#### Organic Substrate and Product Concentrations

Organic substrates, namely, nitrobenzene and the three nitrotoluene isomers, as well as nitrobenzyl alcohols and catecholic products were quantified by high-performance liquid chromatography, whereas NO_2_^–^ was measured photometrically. All procedures have been described previously.^24,25^

#### Stable Isotope Analyses

The 12 mL vials were prepared for analysis of ^18^O/^16^O ratios in O_2_ by creating a 3 mL headspace filled with N_2_.^24,105,106^ After partitioning of dissolved O_2_ into the headspace, 1000 μL of gaseous sample was withdrawn and injected into a GC/IRMS (Thermo Fisher Scientific) equipped with two sequentially connected PLOT columns (Restek from BGB Analytik; 30 m × 0.32 mm ID, 30 μm film thickness). Instrument parameters and calibrations followed methods described in Bopp et al.^107^ Carbon isotope ratios (^13^C/^12^C) of organic substrates were analyzed on a GC/IRMS after solid phase microextraction as described in earlier works.^106,108^

### Data Evaluation

#### Reaction Stoichiometries

Substrate transformation and product generation were quantified in terms of reaction stoichiometries, which were normalized to the amount of consumed NADH as shown in eq 1. Stoichiometric coefficients of species *j*, |υ_*j*_|, were calculated through linear regressions of eq 1 for the different concentrations of the nitroaromatic substrate, dissolved O_2_, hydroxylated aromatic product, and NO_2_^–^ obtained from assays with different amounts of added NADH after completion of turnover.
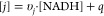
1where |υ_*j*_| is the stoichiometric coefficient of species *j*, [*j*] is the measured molar concentration of the species after complete consumption of NADH, and [NADH] is the nominal concentration of NADH with *q* as the *y*-intercept. Uncertainties of |υ_*j*_| reflect errors arising from standard deviations in the measurements and linear regression analysis and are reported as 95% confidence intervals.

The extent of O_2_ uncoupling, , was determined by linear regression of eq 2.
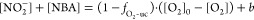
2where [NO_2_^–^] and [NBA] are the concentrations of NO_2_^–^ and nitrobenzylalcohol formed, respectively.  and [O_2_] are the initial and final O_2_ concentrations, respectively. We accounted for the possible constant O_2_ background consumption independent of NADH concentration according to procedures described in Bopp et al.^24^ The corresponding data are shown in Section S2.5 together with a discussion of the sensitivity of  to the accuracy of reaction product quantifications at small substrate turnover.

#### Isotope Effects

Kinetic isotope effects pertinent to the hydroxylation of aromatic carbon, ^13^C-KIE, were determined through non-linear regressions of the observable changes in ^13^C/^12^C ratios versus the fractional amount of residual substrate resulting in C isotope signatures, δ^13^C, and C isotope enrichment factors, ε_C_, according to eqs 3 and 4.
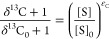
3
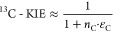
4where δ^13^C and δ^13^C_0_ are the C isotope signatures of the residual substrate after the reaction and its original value, respectively. [S] and [S]_0_ are the final and initial substrate concentrations, respectively, and are documented in Section S2.6. Parameter *n*_C_ is the number of carbon atoms in the substrate to account for the isotopic dilution of the isotope effect assuming an asynchronous hydroxylation mechanism.^101,106^ Non-linear regression fits including the standard deviation of triplicate measurements were carried out in Igor Pro (WaveMetric Inc.).

In a similar procedure, kinetic isotope effects of O_2_ activation, ^18^O-KIE, were derived as average for both O atoms in O_2_ according to eq 5.
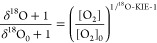
5where [O_2_ ] and [O_2_ ]_0_ are the final and initial dissolved O_2_ concentrations, respectively.

### Computational Analyses of the Protein Structure

#### Generation of Structural Models for 2NTDO and Its Variants

Homology models of wild-type and variants of 2NTDO were generated by submitting the corresponding sequences as a α_3_β_3_ heterohexamer to the AlphaFold-Multimer^109^ while keeping the number of multimer predictions per model to 1. The model structures obtained were aligned to the crystal structure of NDBO (PDB ID: 2BMQ) using PyMOL (v 2.5.2, Schrödinger Inc.). After including hetero atoms, modeled structures were refined applying the Rosetta FastRelax protocol^110,111^ to resolve potential clashes. The model structures of wt 2NTDO are shown exemplarily in Figure S2. All homology model structures were predicted with high confidence and overall template modeling scores (ipTM and pTM scores >0.94).^109^ The homology models of variants closely resemble the wt 2NTDO with all-atom root-mean-square deviations (RMSD) between 0.35 and 0.95 Å (Table S6).

#### Substrate Tunnel Identification and Evaluation of Substrate Transport

CaverDock analyses^112^ were carried out with the structural models obtained using the CaverDock Web Interface^113^ by selecting the catalytic iron from the protein chain C (see Figure S2) as the starting point for tunnel identification and using a minimum probing radius of 0.6 Å while keeping default parameters otherwise (i.e., shell depth of 4, shell radius of 3, clustering threshold of 3.5, a maximal distance of 3, and desired radius of 5). Tunnels for nitroaromatic substrate transport analyses were selected as the highest-priority tunnels according to the likelihood ranking of CaverDock. For O_2_ transport analyses, tunnels were selected based on close proximity to the substrate tunnel and a high CaverDock priority score. Ligand transport analyses were carried out with CaverDock^112^ by applying default parameters, namely, discretization delta (Å) of 0.3 and calculating lower-bound trajectories only.

#### Substrate Docking Studies

Molecular docking was carried out using the homology models described above. The ADFRsuite 1.0 was used to prepare the receptors and ligands with fixed torsion as pdbqt files. AutoDock Vina 1.2.3^114^ was subsequently employed for the molecular docking with a fixed seed of 42, exhaustiveness of 64, box size of 15 × 15 × 15 Å^3^, and the catalytic iron of chain C in the α-subunit as center coordinates (see Figure S2). All other parameters were set to default values, namely, a maximum number of binding modes of 9, a minimum RMSD between output poses of 1, a maximum energy difference between the best and worst binding mode of 3 kcal/mol, and a grid spacing of 0.375 Å. Docking poses were ranked according to the shortest Asn258-NO_2_ bonding distance. Specifically, poses exhibiting the shortest distance were chosen as the most plausible ones based on previous knowledge of H-bonding between nitroaromatic substrates and Asn258.^26,115^

### Bioinformatic Analysis

The National Center for Biotechnology Information protein databases were accessed on 13th of January, 2023. We used BLASTP with default parameters and 2-nitrotoluene dioxygenase (AAB40383.1) from *Acidovorax* sp. strain JS42^95^ as a query sequence against two databases: non-redundant protein sequences (nr) and proteins from WGS metagenomic projects (env_nr). Both databases were updated on 12th of January, 2023. For each, the top 5000 hits were pulled and filtered for significant hits with a bitscore above 250. Working with each data set separately, we aligned protein sequences using Clustal Omega^116^ with default parameters and extracted alignment positions corresponding to variants of interest (M248I, L238V-M248I, I204T, I204A, and I204V). To test for phylogenetic patterns in mutations in each data set, an approximate maximum-likelihood phylogeny was estimated using FastTree v.2.1.11 with default parameters.^117^

## Results and Discussion

### Efficiency of Substrate Oxygenation by Wild-Type 2NTDO and Its Variants

**Figure 1:**
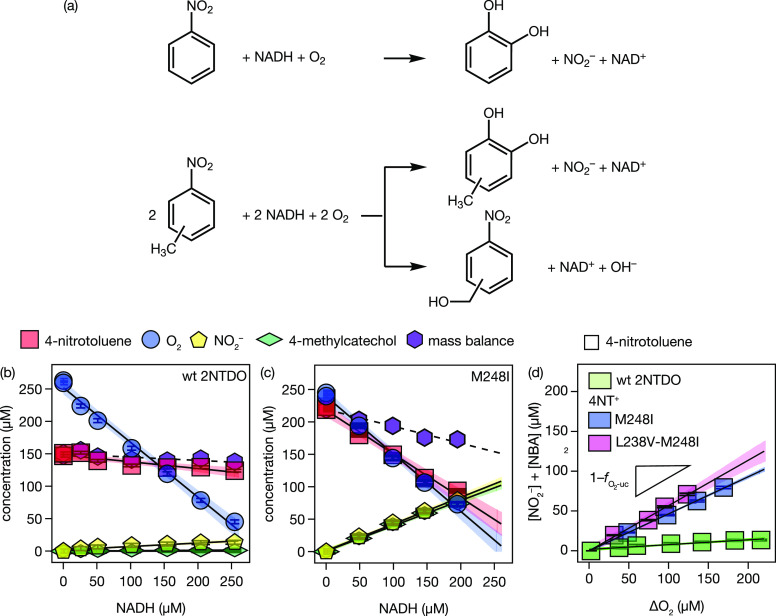
(a) Oxygenation reactions of nitrobenzene and nitrotoluene isomers catalyzed by 2NTDO and its variants. (b) Concentrations of 4-nitrotoluene, dissolved O_2_, organic products, and NO_2_^–^ in enzyme assays of wt 2NTDO after complete consumption of different amounts of NADH.^24^ 4-Nitrobenzylalcohol was not detected in any of the assays, whereas 4-methylcatechol was below the limit of quantification. The black lines and shaded areas represent linear fits with 95% confidence intervals with slopes shown in Table S7. The mass balance includes the concentrations of 4-nitrotoluene, NO_2_^–^, and 4-nitrobenzylalcohol. (c) Experiment with the M248I variant adapted to 4-nitrotoluene. Note that the concentrations of nitrite and 4-nitrocatechol are almost identical whereas 4-nitrobenzylalcohol remained undetected. (d) Efficiency of O_2_ activation as the sum of oxygenation products NO_2_^–^ and nitrobenzylalcohols vs O_2_ consumed, ΔO_2_. The performance of the wild-type oxygenase (2NTDO, green) with 4-nitrotoluene as substrate is compared with variants M248I and L238V-M248I. Lines represent linear fits, their slopes correspond to the oxygenation efficiency  as in eq 2. Shaded areas indicate 95% confidence intervals of linear regressions.

2NTDO and its variants catalyzed the transformation of nitrobenzene and nitrotoluenes to dioxygenated (methyl)-catecholic products, monooxygenated nitrobenzylalcohols, and NO_2_^–^ (Figure 1a). To assess the efficiency of oxygenation vs the unproductive activation of O_2_, we quantified all substrate and product concentrations in enzyme assays at different extents of substrate turnover. In the following, we compare the dioxygenation efficiency of the wt 2NTDO with a variant enzyme arising from a single amino acid substitution in laboratory evolution experiments with 4-nitrotoluene as the substrate (referred to as 4NT^+^ experiments)^95^ for a general illustration of our experimental approach.

Figure 1b shows the consumption of O_2_ and 4-nitrotoluene and concomitant formation of NO_2_^–^ by wt 2NTDO. The 4-methylcatechol product was detected but at concentrations below its quantification limit due to the low substrate turnover. While most of the NADH is used for O_2_ activation (υ_O_2__ = 0.85 ± 0.01 mol/mol NADH, Table 1, entry 4b), only minor amounts of 4-nitrotoluene (υ_S_ = 0.10 ± 0.03 mol/mol NADH, Table 1, entry 4a) are transformed to NO_2_^–^ (υ_NO_2_^–^_ = 0.05 ± 0.01 mol/mol NADH, Table S7). The complete compilation of stoichiometric coefficients for 4NT^+^ experiments obtained with eq 1 is shown in Table S7. The pronounced discrepancy between O_2_ activation and formation of the dioxygenation product was reflected in an almost completely inefficient O_2_ activation by 2NTDO and a fraction of O_2_ uncoupling, , of 0.94 ± 0.01 (eq 2, Table 1, entry 4). Thus, only 6% of the activated O_2_ was used efficiently in the oxygenation of the substrate, whereas the remaining 94% were assigned to inefficient O_2_ activation. This observation concurs with previous ones of low activities of 2NTDO with 4-nitrotoluene.^95^

**Table 1:** Stoichiometric Coefficients, *υ*_*j*_, for O_2_ Activation and Dioxygenation of wt 2NTDO and 4NT^+^ Variants with Nitrobenzene and Nitrotoluenes as Well as the ^13^C-KIEs and ^18^O-KIEs of the Organic Substrates and Dissolved O_2_, Respectively^a^

entry	(co)substrate	*υ*_*j*_^b^	*f*_O___2___-uc_^c^	^18^O-KIE	^13^C-KIE
*2NTDO*^d^					
1a	nitrobenzene	0.47 ± 0.01	0.33 ± 0.02		1.007 ± 0.001
1b	O_2_ (NB)	0.65 ± 0.01		1.015 ± 0.001	
2a	2-nitrotoluene	0.55 ± 0.02	0.02 ± 0.03		1.006 ± 0.002
2b	O_2_ (2-NT)	0.63 ± 0.01		1.016 ± 0.002	
3a	3-nitrotoluene	0.27 ± 0.03	0.84 ± 0.03		1.004 ± 0.001
3b	O_2_ (3-NT)	0.99 ± 0.01		1.018 ± 0.001	
4a	4-nitrotoluene	0.10 ± 0.03	0.94 ± 0.01		1.003 ± 0.001^e^
4b	O_2_ (4-NT)	0.85 ± 0.01		1.021 ± 0.003	
*M248I*					
5a	nitrobenzene	0.83 ± 0.04	0.43 ± 0.02		1.001 ± 0.005
5b	O_2_ (NB)	1.06 ± 0.01		1.013 ± 0.001	
6a	2-nitrotoluene	0.82 ± 0.01	0.05 ± 0.03		1.005 ± 0.005
6b	O_2_ (2-NT)	0.89 ± 0.01		1.012 ± 0.001	
7a	3-nitrotoluene	0.51 ± 0.01	0.73 ± 0.03		1.003 ± 0.004
7b	O_2_ (3-NT)	0.91 ± 0.01		1.017 ± 0.001	
8a	4-nitrotoluene	0.66 ± 0.01	0.53 ± 0.03		1.007 ± 0.006
8b	O_2_ (4-NT)	0.90 ± 0.01		1.019 ± 0.001	
*L238V-M248I*					
9a	nitrobenzene	0.86 ± 0.02	0.39 ± 0.02		1.004 ± 0.003
9b	O_2_ (NB)	1.02 ± 0.01		1.015 ± 0.001	
10a	2-nitrotoluene	0.76 ± 0.01	0.13 ± 0.02		1.005 ± 0.002
10b	O_2_ (2-NT)	0.99 ± 0.01		1.019 ± 0.001	
11a	3-nitrotoluene	0.52 ± 0.01	0.71 ± 0.03		1.002 ± 0.003
11b	O_2_ (3-NT)	0.93 ± 0.01		1.018 ± 0.001	
12a	4-nitrotoluene	0.61 ± 0.01	0.43 ± 0.03		1.004 ± 0.005
12b	O_2_ (4-NT)	0.83 ± 0.01		1.016 ± 0.001	

a The corresponding data for 3NT^+^ experiments are shown in Table S8.

b NADH-normalized stoichiometry of (co)substrate consumption based on eq 1.

c O_2_ uncoupling based on eq 2.

d Reproduced from Bopp et al.^24^

e Reproduced from Pati et al.^108^

The M248I variant of 2NTDO, in contrast, developed the ability to dioxygenate 4-nitrotoluene with much greater efficiency (Figure 1c). While the stoichiometric coefficient of O_2_ consumption, υ_O_2__, remained similar to the wild-type (0.90 ± 0.01 mol/mol NADH, Table 1, entry 8b), consumption of 4-nitrotoluene, υ_S_, increased substantially by almost 7-fold to 0.66 ± 0.01 mol/mol NADH (Table 1, entry 8a). Based on υ_NO_2_^–^_ (0.41 ± 0.01 mol/mol NADH, Table S7), the oxygenation by the M248I variant was 8-fold higher compared to the wt 2NTDO. Thus, the extent of O_2_ uncoupling, , dropped from 94 to 53% for wild-type and the variant, respectively. With increasing NADH concentrations, however, we observed a decrease in the sum of the nitroaromatic substrate and NO_2_^–^ concentrations indicated by mass balance calculations. Similar effects were observed for experiments with 2NTDO and its 4NT^+^ variants, catalyzing 3- and 4-nitrotoluene oxygenation. This observation points to an additional but minor unknown reaction product that did not become evident in NO_2_^–^, methylcatechol, or nitrobenzylalcohol formation and impeded quantification of oxygenation efficiencies by eq 2. An alternative method to determine  based on organic substrate consumption (eq S4) results in M248I -values up to 33% below NO_2_^–^-based values (Table S4). In the following discussion, we will stick to the more conservative estimates based on eq 2 and refer to Section S2.5 for our reasoning. Regardless of this uncertainty, the efficiency of the O_2_ activation by the M248I variant significantly increased compared to that of wt 2NTDO by at least 40%. The comparison of 4-nitrotoluene turnover by wt 2NTDO and the M248I variant illustrates the drastic changes in efficiency of oxygenation caused by a single amino acid substitution. Ju and Parales^95^ showed that an additional substitution (L238V-M248I) evolved directly from the M248I variant. Indeed and as shown in Figure 1d, the double-substituted variant L238V-M248I formed more oxygenation products per O_2_ consumed (ΔO_2_) than both the wild-type and the singly substituted variant M248I (, Table 1, entry 12).

We carried out identical types of experiments for the quantification of oxygenation efficiencies and O_2_ uncoupling for the 3-nitrotoluene adapted variants I204A, I204T, and I204V denoted henceforth as 3-NT^+^ experiments (Figure S3). These enzymes originate from laboratory evolution experiments with *Acidovorax* sp. strain JS42 adapted on 3-nitrotoluene over one month.^96^ In analogy to 4-NT^+^ variants M248I and L238V-M248I, we found that each of these substitutions improved the efficiency of O_2_ activation with 3-nitrotoluene from  of 0.84 ± 0.03 by approximately 25–30% (Figures 1d and S2;  in Table 1, entry 3 vs Table S8, entries 15, 18 and 21), again reflecting an adaptation to the new nitrotoluene substrate isomer.

### Evaluation of Oxygenation Efficiency and O_2_ Uncoupling as a Measure for the Adaptation of Substrate Specificity

We studied the consequences of amino acid substitutions in 2NTDO on the substrate spectra of the enzymes. To that end, we compared oxygenation efficiencies of 2NTDO and five variants from the 4NT^+^ and 3NT^+^ experiments with the four substrates nitrobenzene and the three nitrotoluene isomers.

**Figure 2:**
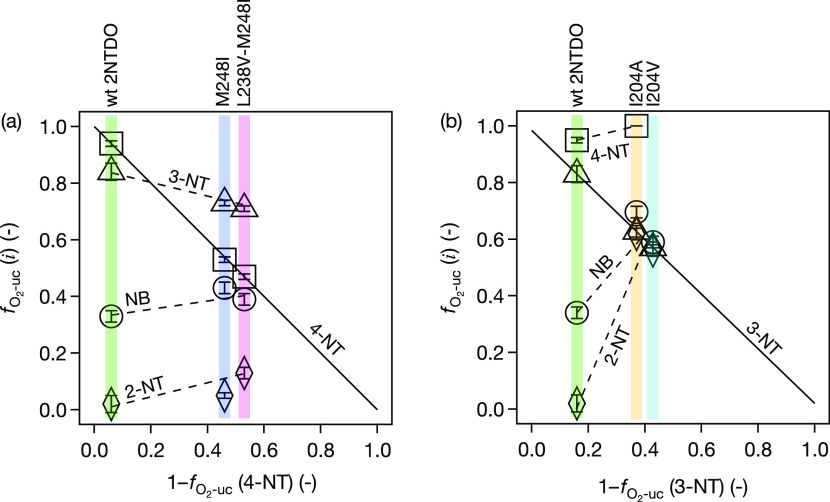
Changes in O_2_ uncoupling, , with nitrobenzene and three nitrotoluene isomers in enzyme assays with wt 2NTDO and variants adapted to (a) 4-nitrotoluene, M248I, L238V-M248I, and (b) 3-nitrotoluene, I204A, I204V. Data for variant I204T is shown in Figure S4.  of the substrates (symbol form) are shown vs the efficiency of the reaction  of the enzymes (colored bars) with the different substrates. The continuous line indicates the uniform relation of  vs  in the target substrate. Error bars represent 95% confidence intervals of O_2_ uncoupling.

Figure 2a shows the fraction of inefficiently activated O_2_, , of the four substrates vs the oxygenation efficiency with 4-nitrotoluene, 1 –  (4-NT), for wt 2NTDO (green), as well as the M248I (blue) and L238V-M248I (pink) variants. The increasing oxygenation efficiency of 4-nitrotoluene in the order 2NTDO, M248I, and L238V-M248I corroborates the observation made by Ju and Parales^95^ who found that the single amino acid substitution at M248I was prerequisite for further adaptation to 4-nitrotoluene through the additional amino acid substitution L238V. The negative 1:1 line in Figure 2a stands for the gradual improvement of the oxygenation efficiency of enzyme variants toward 4-nitrotoluene from 6% with wt 2NTDO to 47 and 57% with M248I and L238V-M248I, respectively. The colored bars designating changes in oxygenation efficiency of the 4NT^+^ experiment in Figure 2a allow one to assess the relatively minor consequence of this adaptation for the other nitroaromatic substrates. While 2-nitrotoluene and nitrobenzene were oxygenated slightly more inefficiently with increases of  by up to 0.10 (dashed lines in Figure 2a), oxygenation of 3-nitrotoluene improved by a similar amount albeit at a substantially higher .

The three amino acid substitutions at position I204 of 2NTDO allowed growth on 3-nitrotoluene.^96^Figures 2b and S3 show the adaptation trends according to the same visual aids used for the 4NT^+^ experiment in Figure 2a. Each variant improved in oxygenation efficiency with 3-nitrotoluene significantly to 0.37 (I204A), 0.41 (I204T), and 0.39 (I204V) compared to the wild-type (0.16). Contrary to the 4NT^+^ experiments, however, the mutations in the 3NT^+^ experiments gave rise to a substantial decrease in oxygenation efficiency for the remainder of the substrates. The -values of nitrotoluene and 2-nitrotoluene increased between 2- and 30-fold to 0.6 and 0.7, respectively, indicating a substantial decline of substrate specificity. 4-Nitrotoluene was a poor substrate for I204 variants and the substrate turnover in these enzyme assays was mostly insufficient to allow reliable quantification of  close to unity. We note that the serial enrichment cultures in the 4NT^+^ experiments ran for 6 months^95^ as compared to only one month in the 3NT^+^ experiments.^96^ Whether this 6-fold shorter adaptation period in the 3NT^+^ experiments is responsible for the more exclusive adaptation toward this one substrate is unclear.

### Structural Analysis of Point Mutations

We explored whether structural changes in the enzymes upon amino acid substitutions relate to the observable changes in oxygenation efficiency from two perspectives. First, we studied if the observed substitutions affected substrate and O_2_ transport from the protein surface to the active site. In addition, we evaluated changes in the binding of nitroaromatic substrates in the active site through molecular docking by constraining the docking modes based on the interaction between Asn258 and the nitro group via hydrogen bonding. The H-bonding interaction with Asn258 is not only a known prerequisite for catalytic activity of nitroarene dioxygenases^26^ but could also modulate the ability of the enzyme to hold the substrate in place for efficient hydroxylation by reactive Fe-oxygen species.^42^

**Figure 3:**
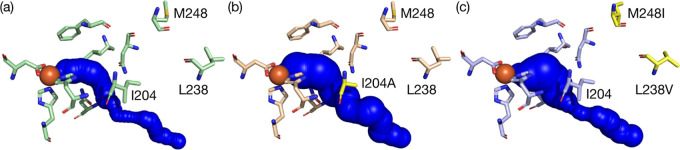
CaverDock analysis of 2NTDO homology models reveals potential tunnels for the transport of the nitroaromatic substrates. Orange spheres indicate the non-heme Fe active site of (a) 2NTDO, (b) I204A, and (c) L238V-M248I.

Following procedures used in previous studies to identify small-molecule tunnels in non-heme Fe enzymes,^104,118−121^ we characterized possible substrate and O_2_ transport tunnels in wt 2NTDO and the five variants using CaverDock^112^ with minimum probing radii of 0.9 and 0.6 Å for substrates and O_2_, respectively. In all homology models, tunnels from the catalytic non-heme Fe site to the surface were localized and could potentially be assigned as nitroaromatic substrate tunnels. Tunnel dimensions and throughput derived with CaverDock are compiled in Table S10 and show lengths between 20 and 23 Å, as well as mean and bottleneck radii of 1.6–1.9 and 1.1–1.5 Å, respectively. Figures 3 and S7 show examples of substrate tunnels for 2NTDO, as well as variants L238V-M248I and I204A from the 4NT^+^ and 3NT^+^ experiments, respectively. As evident from this structural analysis (Figure 3b), I204 is located at the substrate tunnel, whereas residues L238 and M248 are not. The complete list of amino acid residues in the various tunnels is compiled in Table S11. Evidence of I204 acting as a bottleneck residue was obtained from ligand transport analyses. Substrate binding energies calculated with CaverDock along the substrate tunnels are shown in Figure S5. These data indeed revealed lower substrate binding energies at approximately 12 Å distance from the non-heme Fe site through substitutions at the I204 residue.

The same type of CaverDock analysis with a smaller probing radius enabled tentative assignment of O_2_ transport tunnels (Figure S8). While several tunnels can typically be identified which could act as O_2_ transport tunnels (see above and refs^122−124^), a common tunnel structure formed by multiple hydrophobic residues close to the substrate tunnel was identified in all variants. The architecture of the identified tunnels (Table S10) as defined by the mean and bottleneck radii and throughput numbers by CaverDock agrees well with similar analyses of O_2_ tunnel structures in lipoxygenases, another class of non-heme ferrous iron enzymes.^119^

We found that residues L238 and M248 are part of O_2_ transport tunnels identified for wt 2NTDO. However, these residues no longer appear as part of the tunnels that were identified as most likely in the evolved enzyme variants. By contrast, we observed a slightly increased O_2_ tunnel radius and a reduced tunnel curvature for the most likely tunnel in variants M248I and L238V-M248I in comparison to the wild-type. O_2_ binding energies calculated along these O_2_ transport tunnels (Figure S6) are smaller than those for the substrates in substrate transport tunnels (Figure S5). Moreover, differences in O_2_ binding energy are not as pronounced among the variants carrying different amino acid substitutions. In contrast to substrate transport tunnels (Figure S5), bottlenecks for transport of O_2_ are located at a greater distance from the active site and thus in closer proximity to L238 and M248 residues.

This preliminary and, in the absence of molecular dynamics simulations of nitroarene dioxygenases,^115^ purely static evaluation of structural features of O_2_ transport tunnels in 2NTDO and its variants underscores the well-known relevance of tunnels. Tunnels are considered critical for the selectivity of O_2_ transport to the active site but not necessarily for O_2_ activation and reactivity.^104,122,123,123−127^ While our analysis illustrates that amino acid substitutions in the 4NT^+^ and 3NT^+^ experiments occurred at residues that likely affect substrate and O_2_ transport, it is currently unknown how mutations in positions L238, M248, and I204 resulted in a reduction of O_2_ uncoupling. The different locations of substitutions in 4NT^+^ and 3NT^+^ variants appear to point to structurally different modes of adaptation for the two isomers. Changes in the O_2_ transport tunnel in 4NT^+^ variants largely maintained the oxygenation efficiency of other substrates (Figure 2), whereas alterations to the substrate tunnel following adaptation to 3-nitrotoluene decreased oxygenation efficiencies of other substrates. Evidence from engineering O_2_ channels in lipoxygenase^118^ showed that 2-fold larger bottleneck radii increased oxygenation efficiencies and specific protein activities. Here, we do not observe that changes in kinetic parameters associated with O_2_ consumption (e.g., *k*_cat_/*K*_m_-values in Table S12) of wt 2NTDO, variants, and substrates correlate with their O_2_ uncoupling behavior.

**Figure 4:**
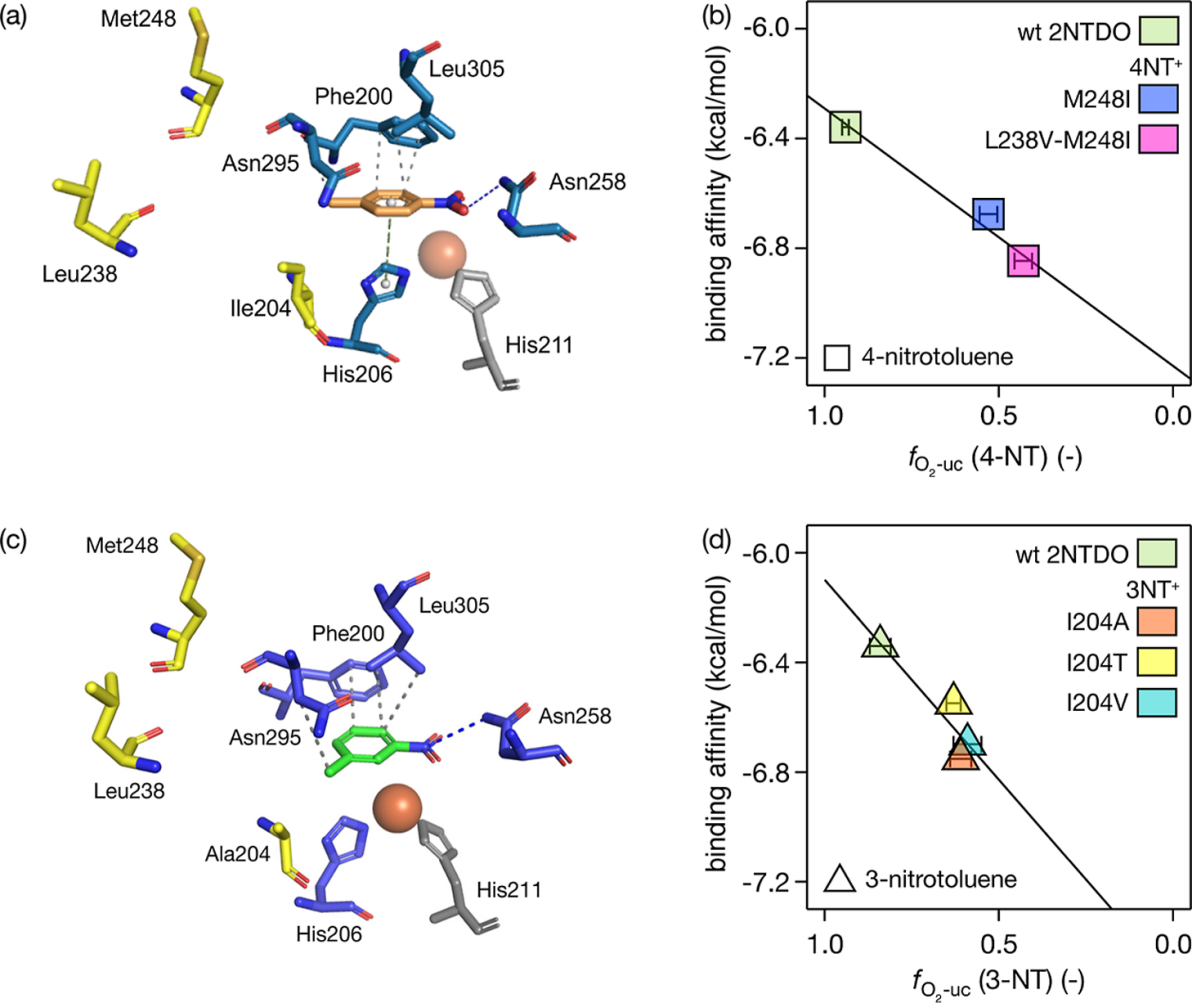
Molecular docking of 4-nitrotoluene (a,b) and 3-nitrotoluene (c,d) into 2NTDO variants. (a,c) Interaction network generated with protein-ligand interaction profiles.^128^ (b,d) Correlation of O_2_ uncoupling, , vs binding affinities of 4- and 3-nitrotoluene obtained from poses with shortest Ar-NO_2_-Asn258 binding distances.

We further studied the consequences of the three point mutations on substrate binding in the active site by evaluating the H-bonding interactions between the aromatic NO_2_ group and residue Asn258 in the various docking poses for each enzyme/variant-substrate combination. Here, we deliberately focus on poses with the shortest Ar-NO_2_-Asn258 binding distance. The comparison of all substrate binding affinities with the extent of O_2_ uncoupling, , is shown in Figure S9. Figure 4 shows the interaction network of docked 4-nitrotoluene into the homology model of variant L238V-M248I and the correlation of binding affinities with -values of this substrate. We found that binding affinities of 4-nitrotoluene and 3-nitrotoluene in wt 2NTDO increase (i.e., become more negative) as a consequence of mutations M248, L238-M248, and I204 (Figure 4b,d). Moreover, the increase of binding affinities correlates approximately linearly with  and supports the conclusion that tighter substrate binding might be advantageous to hold the substrate in place for efficient hydroxylation as invoked for variations of O_2_ uncoupling observed for α-ketoglutarate dependent non-heme ferrous iron oxygenase.^42,43^ However, such correlations were not observed for each enzyme/variant-substrate combination (Figure S9). While selected enzyme variants stemming from the 4NT^+^ and 3NT^+^ experiments show tighter substrate binding compared to wt 2NTDO for some of the tested substrates, systematic trends were observed only for substrates 3- and 4-nitrotoluene (Figure 4). According to common views of enzyme catalysis, one expects tuning of transition state stabilization to occur with a concomitant optimization of the enzymes’ specific activity (i.e., *k*_cat_/*K*_m_)^129^ according to metabolic needs.^29^ A preferred optimization of substrate binding alone, that is lowering of *K*_m_, under the conditions of the adaptation experiments where substrate concentrations exceed *K*_m_ might not necessarily improve catalysis.^130^ Modulation of enzymatic activity through tighter binding of the substrate as a means to avoid O_2_ uncoupling and concomitant production of ROS, by contrast, would appear as a quite meaningful adaptation strategy.

### Catalytic Cycle of 2NTDO Variants

**Figure 5:**
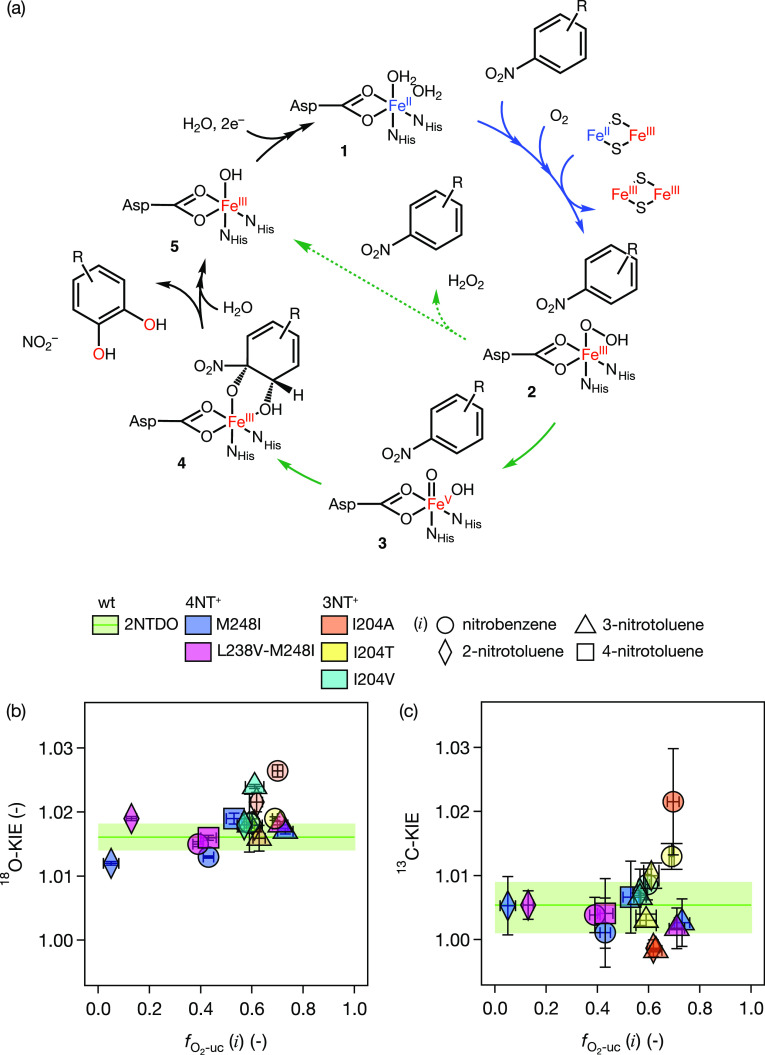
(a) Catalytic cycle of wt 2NTDO and enzyme variants.^24,25^ Upon substrate binding to the 6-coordinate non-heme Fe^II^ resting state **1**, O_2_ is bound concomitant with electron transfer from the reduced Rieske cluster (Fe^II^–Fe^III^). O_2_ uncoupling and release of unreacted substrate and ROS (H_2_O_2_) (dashed arrows) occur from the Fe^III^-oxo-peroxo species **2**. Substrate oxygenation can be catalyzed by species **2** and high-valent Fe-oxo(hydroxo) species **3**. ^18^O kinetic isotope effects (^18^O-KIE) probe for processes involved in O_2_ activation and are indicated with blue arrows. ^13^C-KIEs of nitroaromatic substrate hydroxylation steps are shown with green arrows. (b) ^18^O-KIE values of O_2_ activation in the various enzyme/variant-substrate combinations vs O_2_ uncoupling. Marker shapes stand for substrate type; colors denote enzyme or variant. (c) Corresponding data for ^13^C-KIEs of substrate hydroxylation.

We examined the catalytic cycle of 2NTDO variants shown in Figure 5 and probed for changes in rate-limiting steps of O_2_ activation and substrate hydroxylation upon amino acid substitution by evaluating nitroaromatic substrate and O_2_ kinetic isotope effects (KIEs). Competitive ^18^O and ^13^C kinetic isotope effects of dissolved O_2_ and the nitroaromatic substrate, respectively, were quantified following the methodology of our previous works^24,25,106^ and the data are compiled in Tables 1 and S8. Figure 5b shows the ^18^O-KIEs of O_2_ activation in the presence of nitrobenzene and different nitrotoluene isomers for enzyme variants. ^18^O-KIEs of most variant-substrate combinations ranged from 1.012 to 1.020 with an average value of 1.017 ± 0.003. This outcome is indicative of a kinetic mechanism in which the rate-limiting step of O_2_ activation involves formation of Fe^III^-(hydro)peroxo species^131^ (**2** in Figure 5a). This interpretation is corroborated by the detection of H_2_O_2_ from O_2_ uncoupling in assays of variants (Table S2). Almost identical observations in terms of ^18^O-KIE were made with wt 2NTDO (1.016 ± 0.002)^24^ and another nitroarene dioxygenase^25^ with an even larger set of substrates implying that the mechanism of O_2_ activation remained identical in 2NTDO variants. We do, however, also find three ^18^O-KIE values between 1.022 and 1.026 for oxygenation of nitrobenzene and nitrotoluene isomers by I204 variants which exceed the values of the other ^18^O-KIE in the rest of our data. These values approach theoretical ^18^O equilibrium isotope effects (^18^O-EIEs) that are considered representative of the rate-limiting formation of Fe^IV^=O species (^18^O-EIE of 1.0287).^131^ This mechanism of O_2_ activation is also consistent with reactions of other Rieske oxygenases.^132,133^ Whether this finding hints at changes in O_2_ activation mechanisms in I204 variants is currently unclear. The higher ^18^O-KIE values make up 30% of the data for I204 variants but less than 6% of the approximately 50 ^18^O-KIE values that we have determined so far for nitroarene dioxygenases.^24,25^

^13^C Kinetic isotope effects (^13^C-KIEs) of nitroaromatic substrate hydroxylation were used as probes for changes at later stages of the catalytic cycle in the variants. Specifically, we characterized the timing of substrate hydroxylation vs O_2_ uncoupling (green arrows in Figure 5a). The ^13^C-KIE values for substrate hydroxylation by the different 2NTDO variants were, again, similar to what was observed previously for wt 2NTDO (0.998–1.011, Figure 5c, Tables 1 and S8).^24^ These data indicate a lack of reversibility of species **3** formation, from which hydroxylation occurs with an intrinsic ^13^C-KIE of up to 1.039.^108^ As a consequence, the unreacted substrate released upon O_2_ uncoupling was not subject to any isotope fractionation from reaction **3** → **4** (Figure 5a).^24^ The two exceptions to this interpretation with large ^13^C-KIE values (I204T and I204A of 1.013 ± 0.002 and 1.022 ± 0.008, respectively, Figure 5c) also come from reactions catalyzed by I204 variants. In analogy to the interpretation of ^18^O-KIE data above, these data could be seen as first and thus preliminary evidence for an alteration of the hydroxylation mechanism in enzyme variants. Larger ^13^C-KIE values, by contrast, are not uncommon. Such values are indicative of the slightly different catalytic scheme as observed previously for another nitroarene dioxygenase where the magnitude of C isotope fractionation in the substrate was shown to correlate with the extent of O_2_ uncoupling.^25^

### Comparison of In Vivo Evidence for Adaptation with Oxygenation Efficiency Quantified In Vitro

We hypothesize that O_2_ uncoupling and the ensuing formation of ROS limits the fitness of *Acidovorax* sp. strain JS42 in a substrate-dependent manner, which would also correlate with the adaptation of mutant strains to alternative substrates. To examine O_2_ uncoupling as a measure for the characterization of microbial adaptation processes, we compared the oxygenation efficiencies obtained in vitro (in enzyme assays) with in vivo parameters for microbial activity (as NO_2_^–^ formation rates) and growth (from inverse doubling times). The in vivo parameters were taken from previous experiments with evolved *Acidovorax* strains^95,96^ and are referred to here collectively as NT^+^ strains.

**Figure 6:**
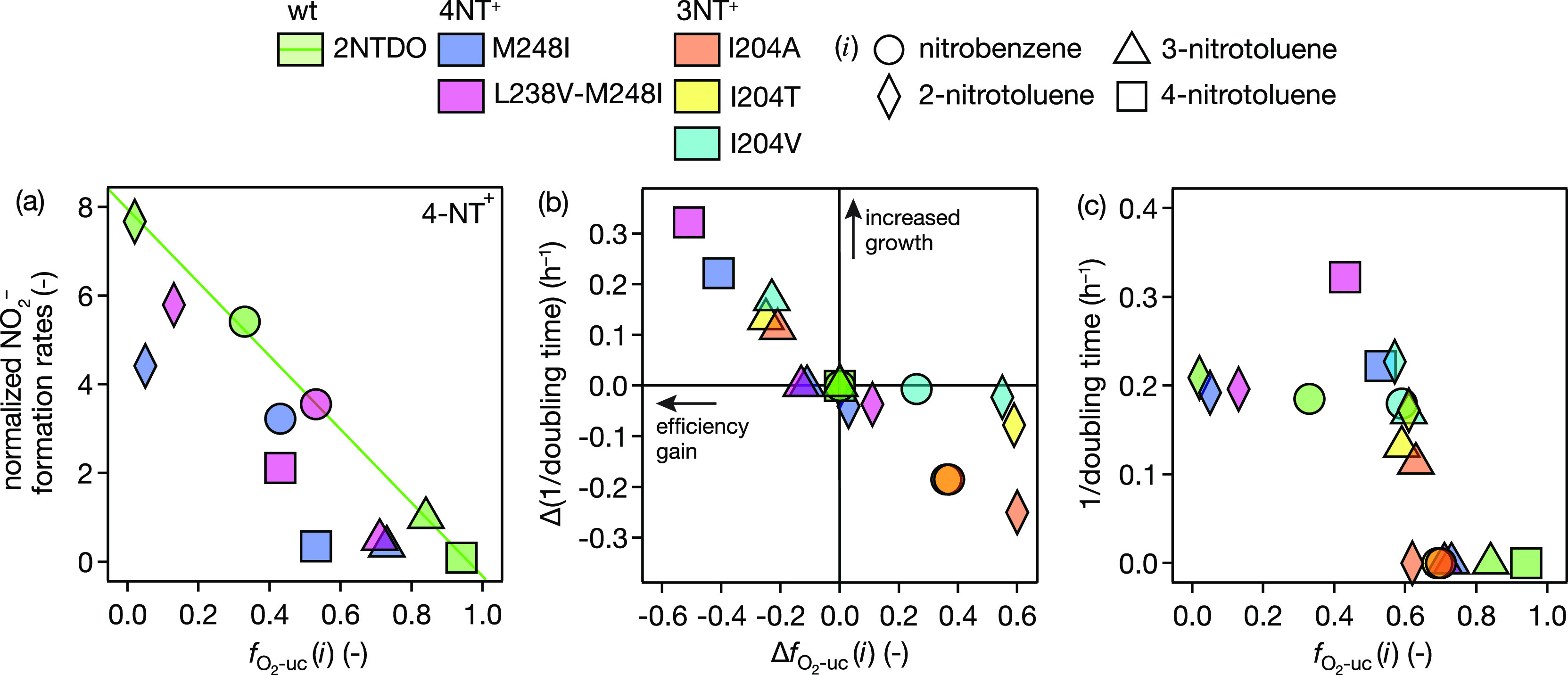
(a) Rates of nitrite formation by *Acidovorax* sp. J42 and evolved 4NT^+^ strains (per mg of protein) normalized to value obtained for 3-nitrotoluene by wt 2NTDO vs  from enzyme assays with wt 2NTDO and variants from 4NT^+^ experiments for 4 substrates. (b) Changes of inverse doubling times, Δ(1/doubling time), of evolved 4NT^+^ and 3NT^+^ strains relative to inverse doubling times determined with *Acidovorax* sp. J42 strain and the 4 substrates vs changes of O_2_ uncoupling, , of variants relative to  obtained with the 4 substrates with wt 2NTDO. (c) Inverse doubling times vs . Note that all data used for *y*-axes originate from whole cell experiments and were obtained from Ju and Parales^95^ and Mahan et al.^96^ No growth was assigned a value of 0 h^–1^.

The comparison of specific in vivo NO_2_^–^ formation rates of *Acidovorax* sp. strain JS42, 4NT^+^, and 3NT^+^ strains expressing 2NTDO variants with the -values determined with the variants are shown in Figures 6a and S10. We observed excellent linear correlations of NO_2_^–^ formation rates determined in vivo with -values for oxygenation of all four substrates by wt 2NTDO derived in vitro, confirming the validity of our comparisons. Favorable amino acid substitutions in wt 2NTDO are expected to lead to increased oxygenation efficiencies (i.e., decrease of ) and thus increased relative NO_2_^–^ formation rates in whole cell systems (and vice versa). The relationship between changes of O_2_ uncoupling and responses in activity, however, was not necessarily linear. The decrease of  of 4-nitrotoluene from 0.94 to 0.43 (Table 1, Figure 6a) became especially evident in the doubly substituted oxygenase variant L238V-M248I but not so much in M248I. This trend implies that the increase in dioxygenation activity and thus NO_2_^–^ formation rate in vivo could have been the consequence of the decrease of O_2_ uncoupling that we quantified in vitro. Deviations from the linear correlation illustrate that additional factors other than the oxygenation efficiency of the variants are relevant for microbial activity, a finding that points to the inherent limitations of assessing substrate specificity exclusively from the activity of the dioxygenase.

Microbial growth quantified here as inverse doubling times^95,96^ represents a more holistic measure of adaptation in vivo because *Acidovorax* sp. strain JS42 utilizes nitroaromatic substrates as the sole source of carbon and energy. Activity of the dioxygenase and its efficiency in oxygenating the substrate are thus expected to be reflected in microbial growth rates. In fact, while *Acidovorax* sp. strain JS42 expressing wt 2NTDO could not grow on 3- and 4-nitrotoluene, prolonged exposure resulted in mutations in the codons for residues M248, L238, and I204 that supported growth of 4NT^+^ and 3NT^+^ strains on the respective substrate (Table S13). We note that some of these mutations can be related to G → T transversions typically hypothesized for ROS-induced modifications of nucleic acids^134^ (Section S3.6). In Figure 6b, growth-related observations are shown as changes of O_2_ uncoupling, , and of inverse doubling times, Δ(1/doubling time), where no growth is assigned a value of 0 h^–1^. Increased growth rates of 3-NT^+^ and 4-NT^+^ strains with their target substrate 3- and 4-nitrotoluene are correlated linearly with their oxygenation efficiency gain (top left quadrant in Figure 6b). Conversely, the loss of efficiency is accompanied by decreased growth rates on non-target substrates (bottom right quadrant of Figure 6b). The most notable exceptions of these trends stem from 3NT^+^ experiments where growth of the evolved strains is hardly affected even if O_2_ uncoupling increases to almost 60% (I204T, I204V with 2-nitrotoluene). These data points suggest that not every increase in O_2_ uncoupling is directly translated into reduced growth in vivo probably due to other adaptations not assessed in the original adaptation studies.

A comparison of absolute inverse doubling times vs  for all strains carrying wt 2NTDO and variants as well as any of the four substrates in Figure 6c hints at approximately 60% of O_2_ uncoupling as important threshold. Regardless of the enzyme (variant)-substrate combination, *Acidovorax* sp. strain JS42 and NT^+^ strains were only able to grow with substrates that resulted in  < 0.63. All tested amino acid substitutions lowered  below this threshold with the target substrate. By contrast,  of enzyme (variant)-substrate combination exceeding this threshold could no longer support growth of the microorganisms. Below  of 0.63, all strains showed quite similar growth rates (i.e., inverse doubling times) suggesting that the consequences of oxidative stress such as enzyme self-hydroxylation and reconfiguration of metabolic fluxes^55,135^ are tolerable and other factors than oxygenation efficiency appear to limit growth to similar doubling times. Oxidative stress from O_2_ uncoupling <63% may thus no longer exert selective pressure for adaptations that decrease O_2_ uncoupling even further. Indeed, our observations hint at adaptations that help handle oxidative stress, as growth rates of complemented *Acidovorax* strains carrying no mutations other than the *ntdAcAd* genes encoding 2NDTO (Figure S11) were generally lower. Our observations show that O_2_ uncoupling above the 63% threshold was indeed limiting the adaptation of *Acidovorax* to new substrates. Both the inefficient use of energy for O_2_ activation and the cost of dealing with elevated ROS levels are detrimental to cell growth. Narrowly confined -values of 2NTDO variants with their target substrates (0.43–0.63, Figure 2) indicate a clear threshold where oxygenation becomes beneficial and other factors determine fitness.

## Conclusions

Environmental microorganisms capable of oxidative biodegradation have been shown to evolve their enzymatic machinery upon exposure to xenobiotic compounds. Their biodegradation makes anthropogenic contaminants available as alternative sources of carbon and energy.^11,38,39,95,96^ To that end, adaptation processes have been postulated that would be initiated by unproductive O_2_ activation concomitant with production of ROS during the first chemical steps of contaminant transformation. Many biochemical processes are indeed necessary to lead from O_2_ uncoupling, ROS release, and ROS scavenging to beneficial mutations that ultimately allow for the expression of oxygenases with improved function. Yet, our work suggests that the changes of the efficiency of substrate oxygenation could hint at the occurrence of this complex adaptation process. Point mutations at the terminal oxygenase of 2NTDO acquired from two independent laboratory evolution experiments both led to variants that enabled evolved strains to grow on alternative substrates concomitant with a substantial efficiency improvement of the oxygenases toward these alternative substrates. Analysis of sequence data also confirms the relevance of these variants in an environmental context through the presence of all variants in environmental sequences with relatively high natural abundances and taxonomic distributions among 2NTDO homologues (Figure S12).

The observation that these 2NTDO point mutations could be associated with substrate and O_2_ transport tunnels furthermore supports the notion that an enzymatic reaction can become more efficient while maintaining the kinetic mechanism of enzyme catalysis. The same structural elements have, in fact, been identified as hotspots for engineering Rieske oxygenases not only toward altering C–H hydroxylation selectivity^92−94^ but also for transformation of alternative substrates.^66^ The observation that evolving biodegradation potential and rational engineering can be related to amino acid substitution at the same structural entities offers a promising avenue to assign change in the Rieske oxygenase structure to its function. This kind of insight will be critical for making more causal connections between structural features of this class of enzymes and the biodegradability of organic soil and water contaminants. We note, however, that it remains to be elucidated how modifications in substrate and O_2_ transport tunnels would modulate substrate binding affinities in the active site and the oxygenation efficiencies of target substrates only.

## Supporting Information Available

Biological and chemical materials used, descriptions of experimental, analytical, and computational procedures for protein purification, enzyme assays, chemical, and isotopic analyses and protein structure evaluation, additional results on substrate oxygenation efficiency, tunnel identification, substrate docking, and enzyme kinetics; sequence alignment and similarities scores for nitrobenzene dioxygenase and wt 2NTDO; and comparison of oxygenation efficiencies with fitness parameters and analysis of taxonomic distribution of 2NTDO homologues (PDF)
