# Supplementary material for: Elucidating the Role of O2 Uncoupling for the Adaptation of Bacterial Biodegradation Reactions Catalyzed by Rieske Oxygenases
Source: ACS Environ Au. 2024 May 14;4(4):204–18. doi: 10.1021/acsenvironau.4c00016 (PMC11258757; doi:10.1021/acsenvironau.4c00016)
Supplement: Supplementary file 1 — vg4c00016_si_001.pdf [file vg4c00016_si_001.pdf]

Supporting Information

Elucidating the Role of O_2 Uncoupling for the Adaptation of Bacterial Biodegradation Reactions Catalyzed by Rieske Oxygenases

Charlotte E. Bopp,[1,2] Nora M. Bernet,[1,2] Fabian Meyer,[3] Riyaz Khan[1], Serina L. Robinson,[1,2] Hans-Peter E. Kohler,[1,2] Rebecca Buller,[3] and Thomas B. Hofstetter[1,2*]

[1]Eawag, Swiss Federal Institute of Aquatic Science and Technology, CH-8600 Dübendorf, Switzerland, [2]Institute of Biogeochemistry and Pollutant Dynamics (IBP), ETH Zürich, CH-8092 Zürich, Switzerland; [3]Competence Center for Biocatalysis, Institute of Chemistry and Biotechnology, Zürich University of Applied Sciences, 8820 Wädenswil, Switzerland

[*]Corresponding author: thomas.hofstetter@eawag.ch

28 Pages, 12 Figures, 13 Tables

Contents

S1 Chemicals and Biological Materials **3**

S2 Experimental, Analytical, and Computational Procedures **4**
S2.1 Site-directed mutagenesis . . . 4
S2.2 Sequence alignment and similarity scores of nitroarene oxygenases . . . 5
S2.3 Kinetics of enzymatic O_2 consumption . . . 6
S2.4 Kinetics of substrate oxygenation . . . 6
S2.5 H_2O_2 quantification . . . 7
S2.6 Quantification of O_2 uncoupling . . . 7
S2.6.1 Background consumption of O_2 . . . 7
S2.6.2 Sensitivity of $f_{O_2\text{-uc}}$ quantification to incomplete mass balances . . . 8
S2.7 $^{13}C/^{12}C$ ratio analysis in substrates with limited turnover . . . 10
S2.8 Homology models for computational analysis of protein structure . . . 12

S3 Additional Results **13**
S3.1 Efficiency of substrate oxygenation by 2NTDO: $4NT^+$ experiments . . . 13
S3.2 Efficiency of substrate oxygenation by 2NTDO: $3NT^+$ experiments . . . 15
S3.3 Computational evaluation of protein structure . . . 18
S3.3.1 Substrate tunnel identification . . . 18
S3.3.2 Docking studies . . . 23
S3.4 Enzyme Kinetics . . . 24
S3.5 Qualitative comparison of fitness parameters of whole cell systems . . . 25
S3.6 Significance of oxidative stress-induced oxidation of nucleic acids . . . 25
S3.7 Comparison with *in vivo* evidence for adaptation . . . 26
S3.8 Natural occurrence of 2NTDO homologs . . . 26

S1 Chemicals and Biological Materials

The materials used in this work are identical to the ones detailed in Bopp et al.[1] and are reproduced here.

The following were used as substrates in enzyme assays and used as received. From Sigma-Aldrich (Buchs SG, Switzerland) or Merck (Schaffhausen, Switzerland), we purchased nitrobenzene (99%), 2-nitrotoluene (99%), 3-nitrotoluene (99%), 2-, 3-, 4-chloronitrobenzene (99%), 2-, 3-, 4-fluoronitrobenzene (99%), 2-nitrophenol (99%), 2,6-dinitrotoluene (98%), 2,4-dinitrotoluene (98%), benzoic acid (99%). 4-nitrotoluene (98%) was purchased from Fluka. Catechol (99%), 3- and 4-methylcatechol (95%), 3- and 4-chlorocatechol, 3- and 4-fluorocatechol, 2-, 3-, and 4-nitrobenzylalcohols, and sodium nitrite ($NaNO_2$, 99%) were used as reference compounds to quantify reaction products. 4-Morpholine-ethanesulfonic acid monohydrate (MES, 99%) and potassium phosphate monobasic (KH_2PO_4, 99.5%) were used as buffers and pH was adjusted with sodium hydroxide (NaOH, 99%) and hydrochloric acid (HCl, 32 %). In addition, β-nicotinamide adenine dinucleotide reduced disodium salt (NADH, 97%), peroxidase from horseradish (type VI, 250 pyrogallol units/mg), hydrogen peroxide (H_2O_2, 30%), 4-methoxyaniline, Ampliflu (98%) and ammonium ferrous sulfate hexahydrate ($(NH_4)_2Fe(SO_4)_2$, 99%) were used in enzyme assays. *N*-(1-naphthyl)ethylenediamine (NED, 99%), sulfanilamide (99%), and sodium sulfite anhydrous ($NaSO_3$, 97%) were used for quantitative analyses of $NO_2{}^-$. Methanol (LC/MS grade, 99.99%) was purchased from Fisher Scientific (Reinach, Switzerland). He (99.999%), N_2 (99.999%), and O_2 (99.9995%) gases were from Carbagas (Rümlang, Switzerland). Aqueous solutions were prepared in nanopure water (18.2 MΩ· cm, Barnstead NANOpure Diamond Water Purification System). BTGED buffer containing 50 mM Bis-Tris (Sigma-Aldrich, 98%), 5% v/v glycerol (Sigma-Aldrich, 99%), 5% v/v ethanol (Merck, 99.9%), and 1 mM dithiothreitol (Fisher Scientific, 99%) was used for protein purification.

S2 Experimental, Analytical, and Computational Procedures

S2.1 Site-directed mutagenesis

Table S1 lists the primers used for site-directed mutagenesis of pDTG800[2] to obtain plasmids corresponding to pKMM32, pKMM33, and pKMM35 expressing I204V, I204T, and I204A, respectively.[3]

Table S1 List of oligonucleotide primers used in this study.

Plasmid	Variant	Sequence (5'–3')[a]	Description
pKMM35	I204A	CTTTGTAGGTGACGCCTACCACGTTG	forward
		TTTTCCGCAAAAGGCTTCCAGTTGGC	reverse
pKMM33	I204T	CTTTGTAGGTGACACCTACCACGTTG	forward
		TTTTCCGCAAAAGGCTTCCAGTTGGC	reverse
pKMM32	I204V	CTTTGTAGGTGACGTCTACCACGTTG	forward
		TTTTCCGCAAAAGGCTTCCAGTTGGC	reverse

[a] Changed codons are underlined.

S2.2 Sequence alignment and similarity scores of nitroarene oxygenases

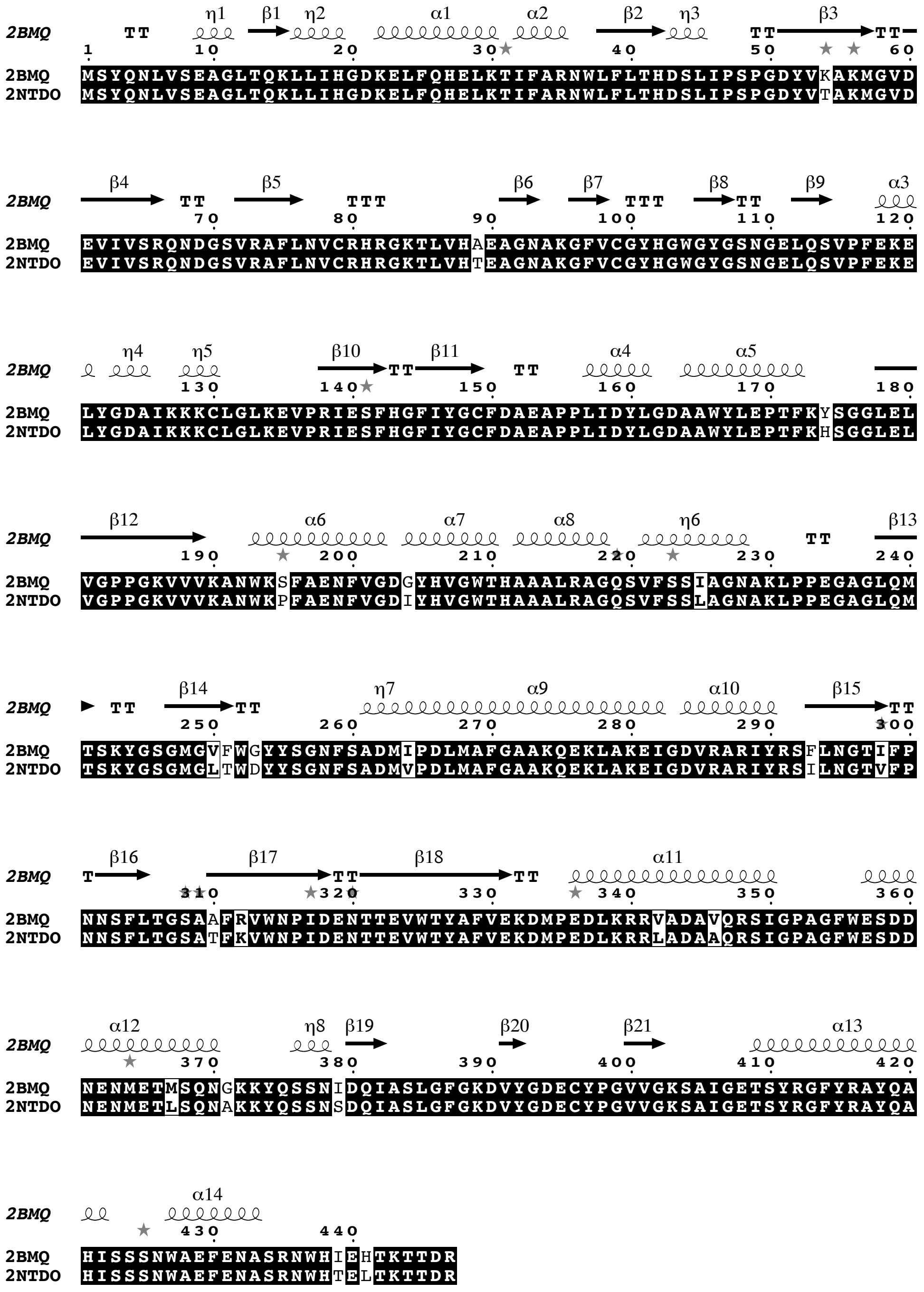

Figure S1 Alignment of the Comamonas sp. JS765 nitrobenzene dioxygenase NBDO α-subunit of PDB ID 2BMQ with the 2NTDO α-subunit from *Acidovorax* sp. strain JS42 (AAB40383.1). The sequence alignment was prepared using Clustal Omega v1.2.4[4] and the secondary structure overlay was added using ESPript v3.0.[5] The protein sequences of NBDO and 2NTDO share 95.3% amino acid identity.

S2.3 Kinetics of enzymatic O_2 consumption

The kinetics of O_2 consumption were determined in 2-mL crimp vials filled completely with enzyme assay containing slightly modified concentrations to prevent anything but O_2 availability limiting turnover with 0.3 μM reductase, 3.6 μM ferredoxin, 0.15 μM oxygenase, 500 μM $(NH_4)_2Fe(SO_4)_2$, and an excess of nitroaromatic substrate (1 mM). We initiated the reaction by adding NADH from a 100 mM stock solution (final concentration 1 mM) and monitored O_2 consumption until complete consumption (250 μM). Initial rates of O_2 consumption, $\nu^i_{0,\mathrm{O_2}}$, were determined from continuous measurements of dissolved O_2 concentration, $c_{\mathrm{O_2}}$, during the first minute after NADH addition.

We obtained ν^i_{max} and K^i_{m} of O_2 consumption from the continuous measurement of O_2 concentrations ($c_{\mathrm{O_2}}$). At each time-point, the rate of O_2 consumption ($\nu^i_{\mathrm{O_2}}$) was calculated as the derivative of measured $c_{\mathrm{O_2}}$ vs. time (i.e., $\Delta[\mathrm{O2}]/\Delta t$). To estimate ν^i_{max} and K^i_{m}, we used non-linear least square regression according to equation S1 with the derived $\nu^i_{\mathrm{O_2}}$ and measured $c^i_{\mathrm{O_2}}$ values.

$$\nu^i_{\mathrm{O_2}} = \frac{\nu^i_{\mathrm{max}} \cdot c^i_{\mathrm{O_2}}}{K^i_{\mathrm{m}} + c^i_{\mathrm{O_2}}} = \frac{k^i_{\mathrm{cat}} \cdot \mathrm{E_0} \cdot c_{\mathrm{O_2}}}{K^i_{\mathrm{m}} + c^i_{\mathrm{O_2}}} \tag{S1}$$

S2.4 Kinetics of substrate oxygenation

The substrate oxygenation kinetics were determined from $NO_2{}^-$ formation of selected enzyme-substrate combinations. Experiments were performed in triplicate in 1.5 mL plastic tubes containing 0.5 mL MES buffer (50 mM, pH 6.8) with 0.3 μM reductase, 3.6 μM ferredoxin, 0.15 μM oxygenase, and 500 μM $(NH_4)_2Fe(SO_4)_2$ at 6 different initial substrate concentrations ranging from 10 to 300 μM. We initiated the reaction by the addition of 500 μM NADH and withdrew 100 μL samples after 20, 30, 40, and 50 seconds. Quenching of the reaction and $NO_2{}^-$ quantification were achieved by mixing the sample with 200 μL of sulfanilamide (10 g L^{-1} in 1.5 M HCl) followed by the addition of 200 μL of *N*-(1-naphthyl)ethylenediamine dihydrochloride (1 g L^{-1} in 1.5 M HCl). $NO_2{}^-$ was quantified photometrically at 540 nm[6].

To determine maximum rates (ν^i_{max}) and Michaelis constants (K^i_{m}) of $NO_2{}^-$ formation in the presence of different substrates, i, we performed a non-linear least square regression according to equation S2,

$$\nu^i_{0,\mathrm{NO_2^-}} = \frac{\nu^i_{\mathrm{max}} \cdot c^i_0}{K^i_{\mathrm{m}} + c^i_0} = \frac{k^i_{\mathrm{cat}} \cdot \mathrm{E_0} \cdot c^i_0}{K^i_{\mathrm{m}} + c^i_0} \tag{S2}$$

where $\nu^i_{0,\mathrm{NO_2^-}}$ is the initial rate of $NO_2{}^-$ formation, c^i_0 is the nominal initial concentration of the substrate, k^i_{cat} is the observable first-order rate constant, and $\mathrm{E_0}$ is the nominal concentration of active sites in the enzyme, corresponding to 3 mol per mol of oxygenase.

S2.5 H_2O_2 quantification

We quantified H_2O_2 concentrations in enzyme assays based on horse radish peroxidase catalyzed turnover of *p*-methoxyanilin. The samples correspond to the maximum turnover samples also used in the determination of stoichiometries and kinetic isotope effects. In Table S2, we present the results for maximum H_2O_2 formation detected in single enzyme assays.

Table S2 Results of H_2O_2 concentration assays. Each entry corresponds to the concentrations in the enzyme with maximum H_2O_2 formation. From the O_2 consumed, ΔO_2, and oxygenation products formed, NO_2^- and nitrobenzylalcohol (NBA), the $f_{O_2\text{-uc}}$ for a single assay is determined. Note that this procedure does not allow for exclusion of background consumption of O_2 (Section S2.6.1).

Substrate	ΔO_2 (μM)	NO_2^- (μM)	NBA (μM)	$f_{O_2\text{-uc}}$ [a]	H_2O_2 (μM)	$H_2O_2/\Delta O_2$	$H_2O_2/f_{O_2\text{-uc}}$ [a] (μM)
L238V-M248I							
nitrobenzene	137	66.1	n.a.[b]	0.52	96.5	0.70	1.35
2-nitrotoluene	171	122	6.01	0.25	41.4	0.24	0.96
I204A							
nitrobenzene	92.2	25.2	n.a.[b]	0.73	10.7	0.12	0.16
2-nitrotoluene	132	16.7	22.9	0.70	2.12	0.02	0.03
3-nitrotoluene	161	59.2	1.05	0.63	12.1	0.08	0.13
4-nitrotoluene	28.9	3.82	0	0.87	6.41	0.22	0.25
I204T							
nitrobenzene	131	39.9	n.a.[b]	0.70	30.9	0.24	0.34
2-nitrotoluene	149	45.5	16.6	0.58	18.1	0.12	0.21
3-nitrotoluene	130	52.1	1.50	0.59	2.56	0.02	0.03
I204V							
nitrobenzene	116	51.6	n.a.[b]	0.56	13.4	0.12	0.21
2-nitrotoluene	155	81.9	3.63	0.45	16.3	0.10	0.22
3-nitrotoluene	70.6	30.1	1.67	0.55	3.09	0.04	0.07

[a] Determined for single assay as $f_{O_2\text{-uc}} = 1 - ((NO_2^- + [NBA])/\Delta O_2)$; [b] n.a. = not applicable.

S2.6 Quantification of O_2 uncoupling

S2.6.1 Background consumption of O_2

As previously discussed in Bopp et al.[1], we corrected for background consumption of O_2 according to equation 2 of the main manuscript.

$$[O_2]^{bg} = -\frac{b}{(1 - f_{O_2\text{-uc}})} \tag{S3}$$

where $[O_2]^{bg}$ is the concentration of O_2 consumed by processes other than dioxygenation and

b is the intercept of the linear regression. An overview of $[O_2]^{bg}$ in all experiments is given in Table S3.

S2.6.2 Sensitivity of $f_{O_2\text{-uc}}$ quantification to incomplete substrate and oxygen mass balances

It has previously been observed by Pati et al.[7] that reactions of 2NTDO with 3- and 4-nitrotoluene result in an incomplete mass balance. It was hypothesized that this is due to an unknown reaction product that is not evident through ${NO_2}^-$ formation. Accordingly, substrate consumption exceeded detectable product formation in cases listed in Table S4. Thus, values of $f_{O_2\text{-uc}}$ may underestimate the true extent of O_2 uncoupling as activated oxygen may have reacted in an unaccounted oxygenation reaction.

One example is the documented formation of 2-methyl-5-nitrophenol in reactions of 2NTDO with 4-nitrotoluene in whole cell assays.[7] Previously, we did not observe any formation of the monooxygenated product in purified enzyme assays.[1] However, due to the high extent of O_2 uncoupling and the lower activity of purified enzyme compared to the whole cell assay, turnover documented in Bopp et al.[1] was only 20 μM compared to roughly 500 μM in Pati et al.[7] and 2-methyl-5-nitrophenol was likely below detection limit. In contrast, the two $4NT^+$ variants, M248I and L238V-M248I, catalyzed turnover of 120 μM and 160 μM 4-nitrotoluene, respectively. We did not monitor for 2-methyl-5-nitrophenol during the HPLC measurement of these experiments. However, peaks that are consistent with 2-methyl-5-nitrophenol UV/Vis spectrum eluted at reasonable retention times (data not shown). Unfortunately, a quantitative evaluation was not possible but rough estimates based on previous calibrations suggest that 2-methyl-5-nitrophenol concentrations would not significantly alter the discrepancy to substrate consumption.

Table S3 Fraction of inefficiently activated, that is uncoupled O_2, $f_{O_2\text{-uc}}$, in assays containing 2NTDO and adapted variants. b is the y-intercept from equation 2 of the main manuscript used for calculation of the O_2 background consumption $[O_2]^{bg}$ with equation S3.[a]

Substrate	n[b]	$f_{O_2\text{-uc}}$[c]	b (μM)	$[O_2]^{bg}$ (μM)
M248I				
nitrobenzene	3	0.43±0.02	−8.6±7.7	15.2±14.1
2-nitrotoluene	4	0.05±0.03	−19.2±5.9	20.1±6.8
3-nitrotoluene	4	0.73±0.03	−1.2±7.2	4.4±26.9
4-nitrotoluene	4	0.53±0.03	−2.9±7.9	6.2±17.4
L238V-M248I				
nitrobenzene	3	0.39±0.02	−17.8±8.9	29.0±15.3
2-nitrotoluene	4	0.13±0.02	−21.2±6.0	24.4±7.6
3-nitrotoluene	4	0.71±0.03	3.6±7.5	−12.4±27.0
4-nitrotoluene	4	0.43±0.03	−21.7±10.4	38.2±20.5
I204A				
nitrobenzene	3	0.70±0.03	−9.27±14.1	30.5±48.7
2-nitrotoluene	5	0.62±0.02	−9.25±4.27	24.2±12.6
3-nitrotoluene	5	0.63±0.02	−1.41±4.70	3.8±12.9
I204T				
nitrobenzene	4	0.69±0.01	0.36±2.61	−1.17±8.58
2-nitrotoluene	4	0.61±0.03	5.05±5.63	5.0±15.3
3-nitrotoluene	4	0.59±0.04	−2.04±7.65	−13.0±19.0
I204V				
nitrobenzene	5	0.59±0.01	3.49±1.52	−8.61±3.92
2-nitrotoluene	5	0.57±0.02	−6.72±4.30	15.5±13.2
3-nitrotoluene	4	0.61±0.03	−4.70±5.45	10.8±10.8

[a] All data represent results from linear regression ± 95% confidence interval; [b] number of available data points;
[c] determined with equation 2 in the main manuscript.

Due to the lack of other unknown peaks and information on additional potential reaction products, we assume that either 3- and 4-nitrotoluene form other products and/or the monooxygenated products not evident in NO_2^- formation, i.e. nitrobenzylalcohol and 2-methyl-5-nitrophenol, react further. At low concentrations, detection of these unknown products in the chromatogram is unlikely. The unknown nature of these reactions results in an uncertainty in the quantification of $f_{O_2\text{-uc}}$. Customarily, efficient oxygenations are quantified by the sum of NO_2^- and, if applicable, nitrobenzylalcohol (equation 2 in the main manuscript).[1,8,9] Due to

the uncertainty in determining the initial concentration, $[S]_0$, quantification based on substrate consumption as in equation S4 is usually less accurate.

$$[S]_0 - [S] = (1 - f_{O_2\text{-uc}}) \cdot ([O_2]_0 - [O_2]) + b \qquad (S4)$$

where [S] and $[O_2]$ are the concentrations of substrate and O_2 determined after the reaction is completed. The cumulative concentrations of catecholic products are consistently found below NO_2^- concentrations,[1,8] due to their lower stability or formation of Fe(II) complexes,[10] making them unsuitable for quantification of $f_{O_2\text{-uc}}$.

Table S4 gives an overview of $f_{O_2\text{-uc}}$ values based on NO_2^- consumption (equation 2 in the main manuscript) or substrate consumption (equation S4). The alternative results for $f_{O_2\text{-uc}}$ values for 2NTDO and $3NT^+$ variants are within 10% of each other which may be due to the relatively low turnover limiting the margin of error caused by unknown reaction products. For $4NT^+$ variants, however, the discrepancies range from 13 to 31%.

Table S4 List of experiments where the sum of NO_2^- and nitrobenzylalcohol concentrations was less than 80% of the consumption of the organic substrate.

Enzyme	Substrate	$f_{O_2\text{-uc}}$ [a]	$f_{O_2\text{-uc}}$ [b]	max turnover[c] μM
2NTDO[d]	3-NT	0.84 ± 0.03	0.80 ± 0.01	30
2NTDO[d]	4-NT	0.94 ± 0.01	0.87 ± 0.01	20
M248I	3-NT	0.73 ± 0.03	0.40 ± 0.01	100
M248I	4-NT	0.53 ± 0.03	0.26 ± 0.01	130
L238V-M248I	3-NT	0.71 ± 0.03	0.49 ± 0.01	100
L238V-M248I	4-NT	0.43 ± 0.03	0.12 ± 0.01	120
I204A	3-NT	0.63 ± 0.02	0.63 ± 0.01	65
I204T	3-NT	0.59 ± 0.04	0.57 ± 0.01	50
I204V	3-NT	0.61 ± 0.03	0.63 ± 0.01	60

[a] Determined with equation 2 from the main manuscript;
[b] determined with equation S4; [c] based on substrate concentrations;
[d] reproduced from Bopp et al.[1].

S2.7 $^{13}C/^{12}C$ ratio analysis in substrates with limited turnover

As previously discussed in Bopp et al.[1], our experimental setup limited the substrate turnover we observed. An overview of initial substrate concentrations, fractional turnover, and maximum observed changes in $\delta^{13}C$, $\Delta\delta^{13}C$, for all experiments are given in Table S5.

Table S5 Maximum substrate consumption, $1 - c/c_0$, of various substrates in assays of 2NTDO and variants, and observed $^{13}C/^{12}C$ fractionation as changes in $\delta^{13}C$. $[S]_0$ is the nominal initial substrate concentration.[a]

Substrate	$[S]_0$ (μM)	$f_{O_2\text{-uc}}$	$1 - c/c_0$	$\Delta\delta^{13}C$ (‰)
M248I				
nitrobenzene	190	0.43±0.02	0.46	0.83
2-nitrotoluene	190	0.05±0.03	0.75	0.80
3-nitrotoluene	200	0.73±0.03	0.5	0.44
4-nitrotoluene	220	0.53±0.03	0.58	0.49
L238V-M248I				
nitrobenzene	200	0.39±0.02	0.69	0.92
2-nitrotoluene	190	0.13±0.02	0.66	0.85
3-nitrotoluene	200	0.71±0.03	0.48	0.14
4-nitrotoluene	220	0.43±0.03	0.53	0.36
I204A				
nitrobenzene	85	0.70±0.03	0.34	1.78
2-nitrotoluene	95	0.62±0.02	0.94	0.28
3-nitrotoluene	90	0.63±0.02	0.81	0.72
I204T				
nitrobenzene	90	0.69±0.01	0.43	2.23
2-nitrotoluene	95	0.61±0.03	0.85	2.56
3-nitrotoluene	105	0.59±0.04	0.51	0.13
I204V				
nitrobenzene	90	0.59±0.01	0.81	2.59
2-nitrotoluene	90	0.57±0.02	0.88	1.87
3-nitrotoluene	95	0.61±0.03	0.67	0.64

[a] Uncertainties represent 95% confidence intervals.

S2.8 Homology models for computational analysis of protein structure

The model $\alpha_3\beta_3$ hetero-hexamer structure of wild-type 2NTDO is shown exemplary in Figure S2. All-atom root mean square deviations (RMSD) in Å obtained with AlphaFold-Multimer and Rosetta FastRelax protocol for wt 2NTDO and its variants are compiled in Table S6.

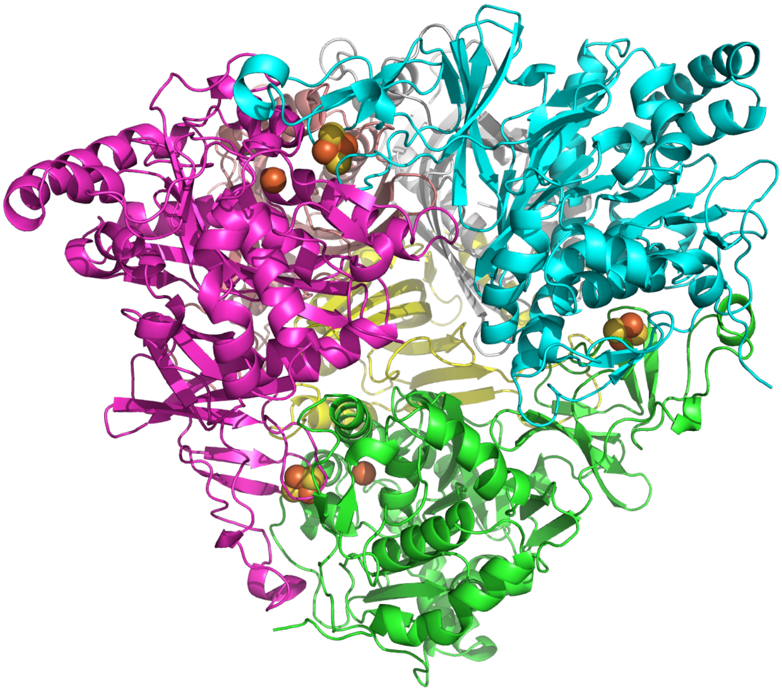

Figure S2 Homology model of $\alpha_3\beta_3$ hetero-hexamer of wild-type 2NTDO generated with AlphaFold-Multimer and refined with the Rosetta FastRelax protocol. The α-subunits consist of chain A (green), chain B (cyan), and chain C (magenta) and the β-subunits consist of chain D (yellow), chain E (salmon) and chain F (grey), while iron and sulfur are depicted as orange and yellow spheres, respectively.

Table S6 All-atom root mean square deviations (RMSD) in Åfor homology models of enzyme variants relative to 2NTDO wild type.

Experiment	Variant	AlphaFold-Multimer	Rosetta FastRelax
$4NT^+$	M248I	0.1549	0.4839
	L238V-M248I	0.0769	0.3491
$3NT^+$	I204A	0.0710	0.3542
	I204T	0.8710	0.9529
	I204V	0.7409	0.8273

S3 Additional Results

S3.1 Efficiency of substrate oxygenation by 2NTDO and variants from 4NT^{+} experiments

(See next page.)

Table S7 Stoichiometric coefficients, $|\upsilon_j|$, of substrate (S) and O_2 consumption as well as formation of NO_2^-, nitrobenzylalcohols (NBA) and catecholic products, catechol (cat), 3-methylcatechol (3-MC), and 4-methylcatechol (4-MC), per nominal concentration of NADH for transformation of nitroaromatic substrates by 2NTDO and $4NT^+$ variants.[a]

**Substrate**	$\lvert\upsilon_S\rvert$	$\lvert\upsilon_{O_2}\rvert$	$\lvert\upsilon_{NO_2^-}\rvert$	$\lvert\upsilon_{cat}\rvert$	$\lvert\upsilon_{3\text{-MC}}\rvert$	$\lvert\upsilon_{4\text{-MC}}\rvert$	$\lvert\upsilon_{NBA}\rvert$
2NTDO							
nitrobenzene[b]	0.47 ± 0.01	0.65 ± 0.01[c]	0.50 ± 0.02	0.42 ± 0.02	n.a.[d]	n.a.	n.a.
2-nitrotoluene[b]	0.55 ± 0.02	0.63 ± 0.01[c]	0.62 ± 0.02	0.63 ± 0.02	0.63 ± 0.02	n.a.	0.03 ± 0.01[e]
3-nitrotoluene[b]	0.27 ± 0.03	0.99 ± 0.01	0.16 ± 0.02	0.10 ± 0.01	0.09 ± 0.01	0.03 ± 0.01	0.02 ± 0.01[f]
4-nitrotoluene[b]	0.10 ± 0.03	0.85 ± 0.01	0.05 ± 0.01	0.01 ± 0.01	n.a.	0.01 ± 0.01	n.d.[g,h]
M248I							
nitrobenzene	0.83 ± 0.4	0.93 ± 0.01[c]	0.50 ± 0.02	0.42 ± 0.02	n.a.	n.a.	n.a.
2-nitrotoluene	0.82 ± 0.01	0.79 ± 0.01[c]	0.73 ± 0.01	0.81 ± 0.02	0.81 ± 0.02	n.a.	0.03 ± 0.01[e]
3-nitrotoluene	0.51 ± 0.01	0.91 ± 0.01[c]	0.23 ± 0.01	0.20 ± 0.03	0.15 ± 0.02	0.05 ± 0.02	0.01 ± 0.01[f]
4-nitrotoluene	0.66 ± 0.01	0.89 ± 0.01[c]	0.41 ± 0.01	0.39 ± 0.01	n.a.	0.39 ± 0.01	n.d.[g]
L238V-M248I							
nitrobenzene	0.86 ± 0.02	0.86 ± 0.01[c]	0.38 ± 0.01	0.76 ± 0.02	n.a.	n.a.	n.a.
2-nitrotoluene	0.76 ± 0.01	0.86 ± 0.01[c]	0.73 ± 0.01	0.77 ± 0.02	0.77 ± 0.02	n.a.	0.01 ± 0.01[e]
3-nitrotoluene	0.52 ± 0.01	0.93 ± 0.01	0.28 ± 0.01	0.29 ± 0.03	0.20 ± 0.02	0.09 ± 0.02	0.04 ± 0.01[f]
4-nitrotoluene	0.61 ± 0.01	0.67 ± 0.01[c]	0.38 ± 0.01	0.37 ± 0.01	n.a.	0.37 ± 0.01	0.01 ± 0.01[g]

[a] Quantification on the basis of equation 2 in the main manuscript, uncertainties correspond to 95%-confidence intervals arising from linear regression analysis; [b] reproduced from Bopp et al. [1];
[c] without O_2 background consumption according to equation S3; [d] n.a. = not applicable; [e] 2-nitrobenzylalcohol;
[f] 3-nitrobenzylalcohol; [g] 4-nitrobenzylalcohol; [h] n.d. = not detected.

S3.2 Efficiency of substrate oxygenation by 2NTDO variants from 3NT$^+$ experiments

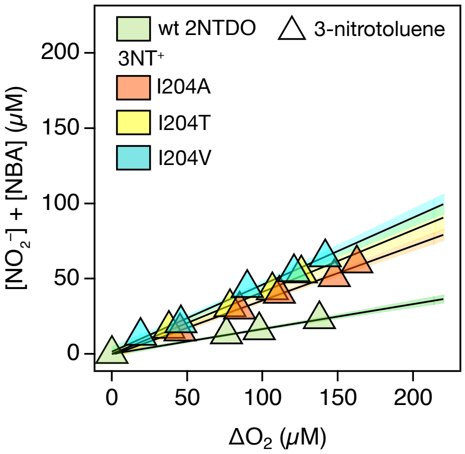

Figure S3 Efficiency of O_2 activation as the sum of oxygenation products NO_2^- and nitrobenzylalcohols vs. O_2 consumed, ΔO_2. Performance of the wild-type oxygenase (2NTDO, green) is compared with I204 variants in transformations of 3-nitrotoluene. Lines represent linear fits, their slopes correspond to the oxygenation efficiency $1-f_{\text{O}_2\text{-uc}}$ as in eq. 2 in the main manuscript. Error bars would be smaller than the marker size and were omitted for better visualization.

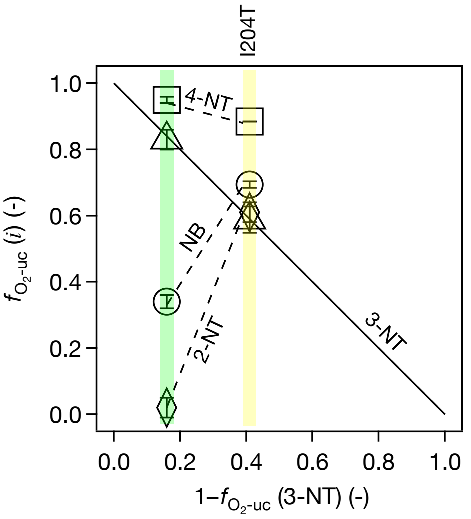

Figure S4 Changes in O_2 uncoupling, $f_{\text{O}_2\text{-uc}}$, with nitrobenzene and three nitrotoluene isomers in enzyme assays with 2NTDO and variant I204T adapted to 3-nitrotoluene. $f_{\text{O}_2\text{-uc}}$ of the substrates (symbol form) are shown vs. the efficiency of the reaction (1-$f_{\text{O}_2\text{-uc}}$) of the enzymes (colored bars) with the target substrate of adaptation. The line indicates the uniform relation of $f_{\text{O}_2\text{-uc}}$ vs. (1-$f_{\text{O}_2\text{-uc}}$) in the target substrate. Error bars represent 95% confidence intervals of O_2 uncoupling and oxygenation efficiency, respectively.

Table S8 Stoichiometric coefficients, v_j, for O_2 activation and dioxygenation of wild-type 2NTDO and 3-NT$^+$ variant enzymes with nitrobenzene and nitrotoluenes as well as the ^{13}C-KIEs and ^{18}O-KIEs of the organic substrates and O_2, respectively.

entry	(co)substrate	v_j [a]	$f_{O_2\text{-uc}}$ [b]	^{18}O-KIE	^{13}C-KIE
I204A					
16a	nitrobenzene	0.12 ± 0.01	0.70 ± 0.03		1.022 ± 0.008
16b	O_2 (NB)	0.38 ± 0.01		1.026 ± 0.001	
17a	2-nitrotoluene	0.29 ± 0.01	0.62 ± 0.02		0.999 ± 0.001
17b	O_2 (2-NT)	0.63 ± 0.01		1.022 ± 0.002	
18a	3-nitrotoluene	0.26 ± 0.01	0.63 ± 0.02		0.998 ± 0.001
18b	O_2 (3-NT)	0.70 ± 0.01		1.016 ± 0.002	
I204T					
13a	nitrobenzene	0.20 ± 0.01	0.69 ± 0.01		1.013 ± 0.002
13b	O_2 (NB)	0.69 ± 0.01		1.019 ± 0.001	
14a	2-nitrotoluene	0.29 ± 0.01	0.61 ± 0.03		1.010 ± 0.001
14b	O_2 (2-NT)	0.83 ± 0.01		1.018 ± 0.001	
15a	3-nitrotoluene	0.43 ± 0.01	0.59 ± 0.04		1.003 ± 0.001
15b	O_2 (3-NT)	0.73 ± 0.01		1.019 ± 0.001	
I204V					
19a	nitrobenzene	0.28 ± 0.01	0.59 ± 0.01		1.009 ± 0.002
19b	O_2 (NB)	0.70 ± 0.01		1.018 ± 0.005	
20a	2-nitrotoluene	0.33 ± 0.01	0.57 ± 0.02		1.007 ± 0.001
20b	O_2 (2-NT)	0.71 ± 0.01		1.018 ± 0.001	
21a	3-nitrotoluene	0.24 ± 0.01	0.61 ± 0.03		1.007 ± 0.004
21b	O_2 (3-NT)	0.71 ± 0.01		1.024 ± 0.001	

[a] NADH-normalized stoichiometry of (co)substrate consumption based on equation 1 in the main manuscript, substrate dihydroxylation is quantified on the basis of NO_2^- concentrations; [b] O_2 uncoupling based on equation 2.

Table S9 Stoichiometric coefficients, $|\upsilon_j|$, of substrate (S) and O_2 consumption as well as formation of NO_2^-, nitrobenzylalcohols (NBA) and catecholic products, catechol (cat), 3-methylcatechol (3-MC), and 4-methylcatechol (4-MC), per nominal concentration of NADH for transformation of nitroaromatic substrates by 3NT$^+$ variants.[a]

**Substrate**	$\lvert\upsilon_S\rvert$	$\lvert\upsilon_{O_2}\rvert$	$\lvert\upsilon_{NO_2^-}\rvert$	$\lvert\upsilon_{cat}\rvert$	$\lvert\upsilon_{3\text{-MC}}\rvert$	$\lvert\upsilon_{4\text{-MC}}\rvert$	$\lvert\upsilon_{NBA}\rvert$
I204A							
nitrobenzene	0.12 ± 0.01	0.51 ± 0.01[c]	0.12 ± 0.01	0.06 ± 0.01	n.a.[b]	n.a.	n.a.
2-nitrotoluene	0.29 ± 0.01	0.56 ± 0.01[c]	0.10 ± 0.01	0.03 ± 0.01	0.03 ± 0.01	n.a.	0.12 ± 0.01[d]
3-nitrotoluene	0.26 ± 0.01	0.70 ± 0.01[c]	0.25 ± 0.01	0.16 ± 0.01	n.d.[e]	0.16 ± 0.01	0.01 ± 0.01[f]
I204T							
nitrobenzene	0.20 ± 0.01	0.69 ± 0.01	0.21 ± 0.01	0.15 ± 0.01	n.a.	n.a.	n.a.
2-nitrotoluene	0.43 ± 0.01	0.81 ± 0.01[c]	0.25 ± 0.01	0.14 ± 0.01	0.14 ± 0.01	n.a.	0.09 ± 0.01[d]
3-nitrotoluene	0.29 ± 0.01	0.73 ± 0.01	0.29 ± 0.01	0.1 ± 0.01	n.d.	0.1 ± 0.01	0.01 ± 0.01[f]
I204V							
nitrobenzene	0.28 ± 0.01	0.70 ± 0.01	0.29 ± 0.01	0.19 ± 0.01	n.a.	n.a.	n.a.
2-nitrotoluene	0.33 ± 0.01	0.57 ± 0.01[c]	0.34 ± 0.01	0.11 ± 0.01	0.11 ± 0.01	n.a.	0.02 ± 0.01[d]
3-nitrotoluene	0.24 ± 0.01	0.68 ± 0.01[c]	0.27 ± 0.01	0.07 ± 0.01	0.01 ± 0.01	0.06 ± 0.01	0.02 ± 0.01[f]

[a] Quantification on the basis of equation 2 in the main manuscript, uncertainties correspond to 95%-confidence intervals arising from linear regression analysis; [b] n.a. = not applicable; [c] without O_2 background consumption according to equation !S3; [d] 2-nitrobenzylalcohol; [e] n.d. = not detected; [f] 3-nitrobenzylalcohol.

S3.3 Computational evaluation of protein structure

S3.3.1 Substrate tunnel identification

Length, mean radius, and bottleneck radius of the substrate and O_2 tunnels, as well as the probability metrics for use of this pathway (throughput) for wild type and its variants obtained by CaverDock are summarized in Table S10. The amino acid residues lining substrate and O_2 tunnels are listed in Table S11 according to the sequence numbering for 2NTDO.

Substrate and O_2 binding energies along substrate and O_2 transport tunnels are shown in Figures S5 and S6. The substrate tunnel identified in variant I204T encounters a distinct problem where inflexible side-chain residues near the surface form an unrealistic bottleneck, resulting in a lower-bound trajectory that is excessively elevated. The three-dimensional structures of substrate transport tunnels for three selected variants are shown in Figure S7. The corresponding structures for O_2 transport for 2NTDO and all variants are compiled in Figure S8.

Table S10 Characterization of substrate and O_2 tunnels identified by CaverDock. Dimensions are given in Å.

	Wild type	Enzyme variants				
Parameter	2NTDO	M248I	L238V-M248I	I204A	I204T	I204V
Substrate						
Length	22.00	23.67	23.24	21.87	24.68	19.62
Mean radius	1.60	1.80	1.70	1.90	1.60	1.90
Bottleneck radius	0.96	1.00	1.04	1.36	0.84	1.37
Throughput	0.34	0.39	0.35	0.46	0.24	0.50
Curvature	1.36	1.34	1.34	1.31	1.34	1.35
O_2						
Length	28.81	27.57	28.42	27.76	26.28	31.36
Mean radius	1.80	1.70	1.60	1.80	1.50	1.60
Bottleneck radius	0.61	0.72	0.65	0.69	0.84	0.70
Throughput	0.19	0.23	0.17	0.23	0.19	0.19
Curvature	1.40	1.35	1.31	1.25	1.63	1.56

Table S11 Amino acid residues lining substrate and O_2 tunnels (residues within 3 Åfrom the tunnel). The residues in substrate tunnels are identical in wt 2NTDO and all variants. Residues in O_2 tunnels fall in 3 groups, (a) wt 2NTDO, (b) M248I, I204A, I204T, I204V, and (c) L238V-M248I.

	Substrate tunnel		O_2 tunnel					
			wt 2NTDO		M248I/I204		L238V-M248I	
Entry	AA	Seq.#	AA	Seq.#	AA	Seq.#	AA	Seq.#
1	Asn	199	Asn	199	Asn	199	Lys	84
2	Phe	200	Phe	200	Phe	200	Thr[a]	85
3	Asp	203	Val[a]	201	Asp[a]	203	Leu[a]	86
4	**Ile**	**204**	Gly	202	Ile[a]	204	Val[a]	87
5	His	206	Asp	203	His	206	His	88
6	Val	207	Ile	204	Val	207	Val[a]	98
7	Gly [a]	208	Tyr [a]	205	His	211	Cys[a]	99
8	His	211	His	206	Gly	218	Gly	100
9	Leu	215	Val	207	Ser	220	Tyr	101
10	Phe	222	His	211	Gly[a]	257	Trp	108
11	Leu	225	**Leu**	**238**	Asn	258	Ala	109
12	Pro	232	Glu	239	Phe	259	Asn	111
13	Pro	233	**Met**[a]	**248**	Ser	260	Arg	148
14	Ala	236	Gly	249	Asp	262	Pro[a]	195
15	Gly	237	Leu[a]	250	Met	263	Phe[a]	196
16	Thr	251	Thr	251	Asn	295	Asn	199
17	Trp	252	Asn	258	Leu	305	Phe	200
18	Asp	253	Ile	293	Ser	308	Asp[a]	203
19	Tyr	254	Asn	295	Thr	310	Ile[a]	204
20	Gly	257	Gly[a]	296	Arg[a]	340	His	206
21	Asn	258	Phe[a]	304	Arg	341	Val	207
22	Ile	293	Leu	305	Leu[a]	342	Trp	209
23	Asn	295	Ser	349	Asp	344	Thr	210
24	Leu	305	Ile	350	Ala	345	His	211
25	Ile	350	Trp	356	Ala	346	Asn	258
26	Trp	356	Glu	357	Gln[a]	347	Asn	295
27	Asp	360	Asp	360	Arg	348	Leu	305
28	Ala	405	Leu	385	Ser	349	Trp	314
29	Ile	406	Gly	386	Ile	350	Ser	349
30			Lys	389	Phe	355	Ile	350
31			Asp	390	Trp	356	Trp	356
32			Val[a]	401	Glu	357	Glu	357
33			Gly	402	Asp	360	Asp	359
34			Lys	403			Asp	360
35			Ser	404			Asn[a]	361
36			Ala	405			Glu	362
37			Ile	406			Asn	363
38			Gly	407			Met	364
39			Glu[a]	408				
40			Thr	409				
41			Arg	412				

[a] Backbone pointing to tunnel.

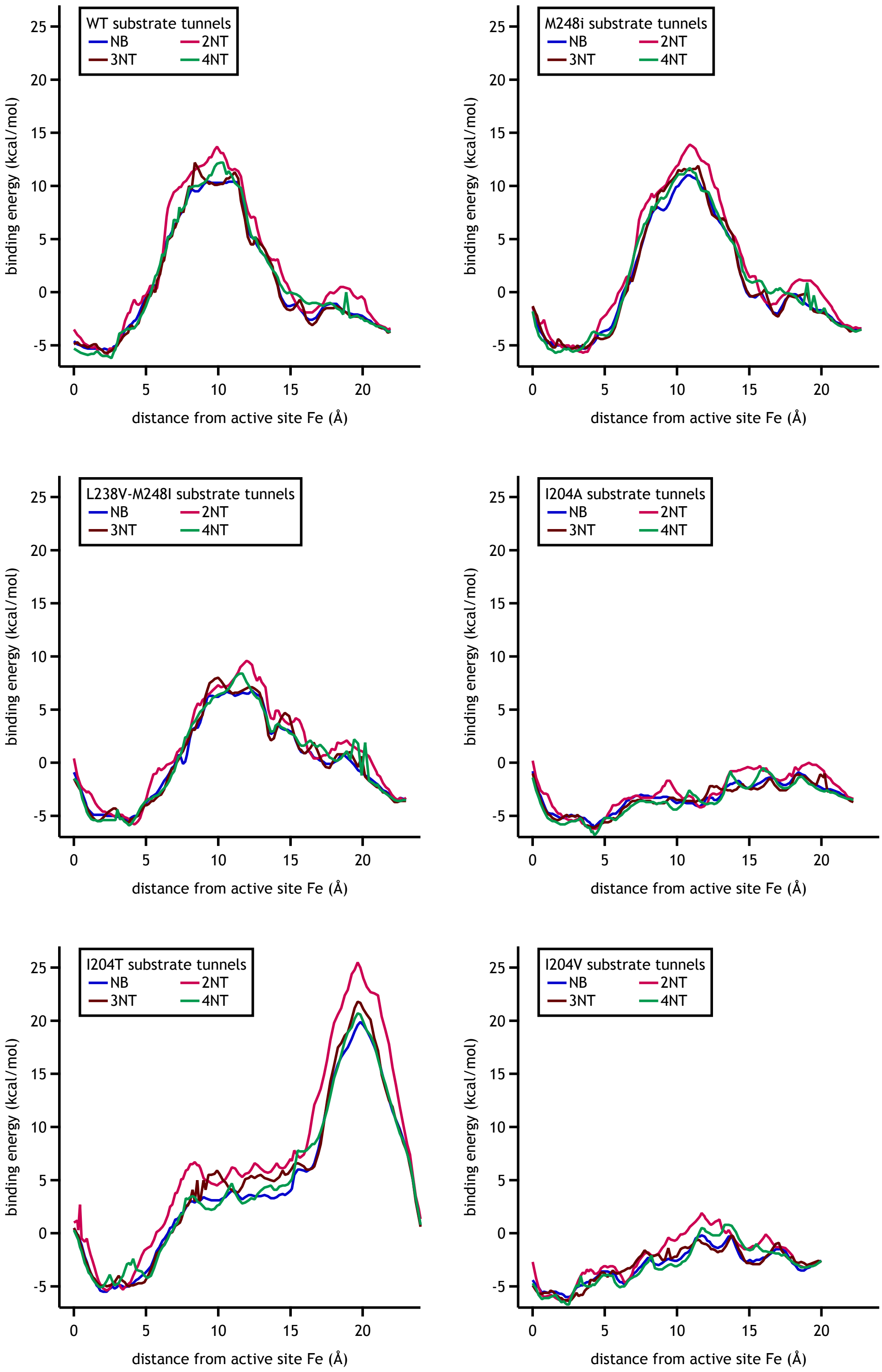

Figure S5 Substrate binding energies for nitroaromatic compound transport along substrate tunnels. Zero distance corresponds to the active site non-heme Fe atom. In the case of variant I204T, the identified tunnel consists of inflexible side-chain residues near the surface resulting in a lower-bound trajectory that is excessively elevated close to the surface. Note the issues with calculations of binding energies calculations for variant I204T mentioned in the text.

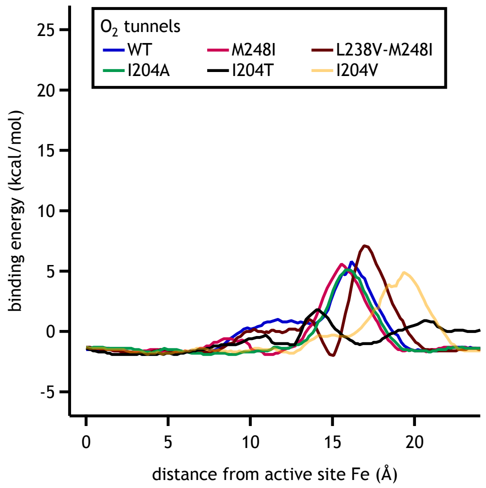

Figure S6 O_2 binding energies along O_2 transport tunnels in wild type 2NTDO and variants. Zero distance corresponds to the active site non-heme Fe atom.

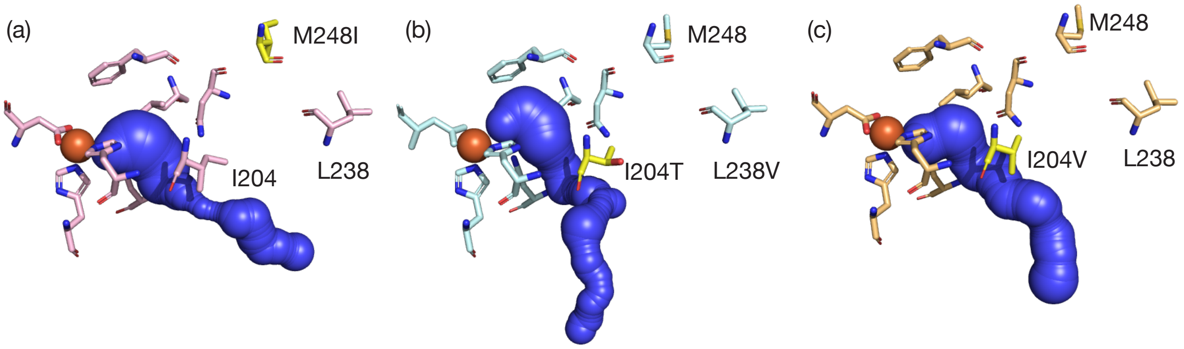

Figure S7 Tunnel structure for nitroaromatic substrates for variants (a) M248I, (b) I204T, (c) I204V.

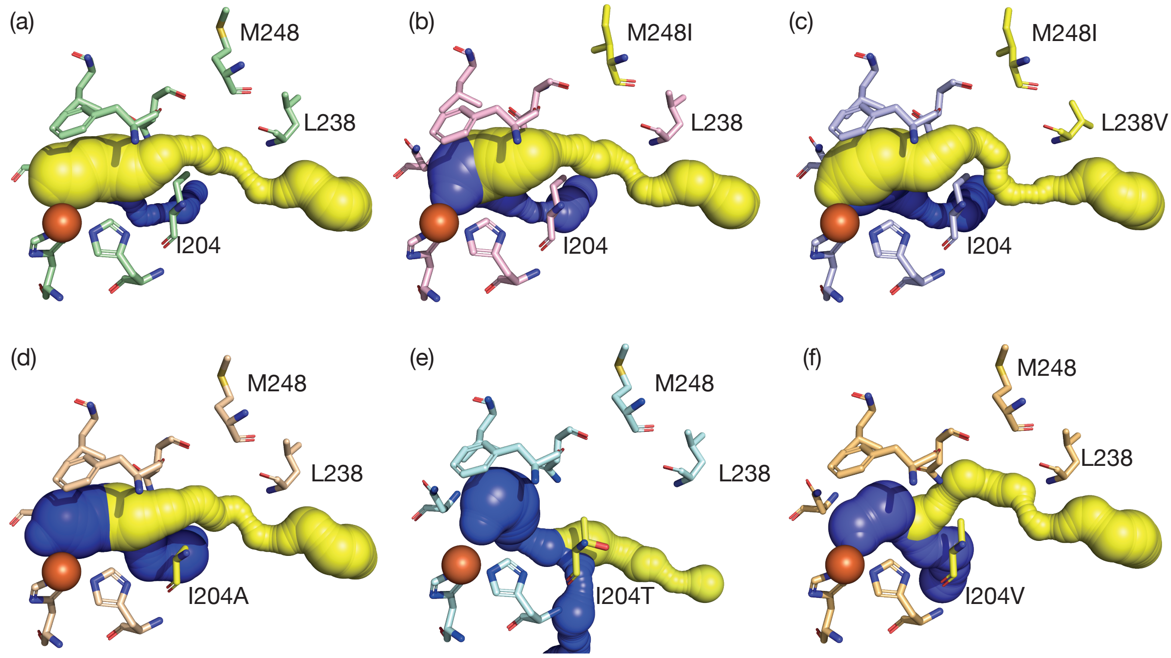

Figure S8 Tunnel structures for O_2 overlayed over nitroaromatic substrates. O_2 transport tunnels are shown in yellow, those for substrate transport are blue. Residues labeled with yellow backbone represent mutated residues in comparison with wt-2NTDO; (a) wt-2NTDO, (b) M248I, (c) L238V-M248I, (d) I204A, (e) I204T, (f) I204V.

S3.3.2 Docking studies

Figure S9 shows the binding affinities of the four substrates, nitrobenzene, 2-nitrotoluene, 3-nitrotoluene, and 4-nitrotoluene for the wt enzyme as well as the variants for poses with shorted H-bonding distance. Bindig affinities (in kcal/mol) are plotted against the extent of O_2 uncoupling, $f_{O_2\text{-uc}}$. The results are grouped according to the two adaptation experiments as $4NT^+$ and $3NT^+$ on top and bottom row, respectively.

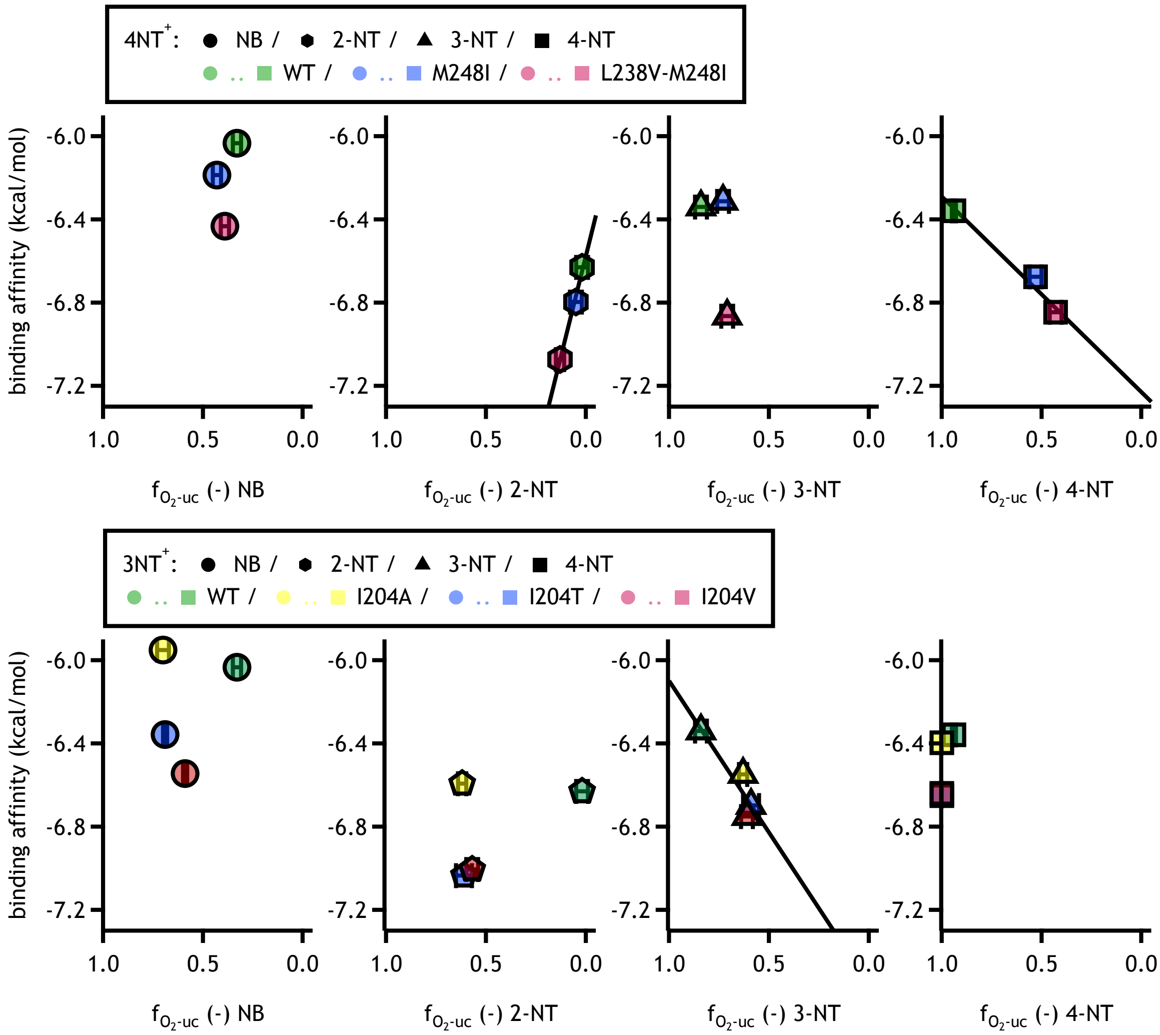

Figure S9 Correlation of binding affinities of nitroaromatic substrates for H-bonding to Asn258 residues in wild type 2NTDO and variant enzymes calculated through molecular docking vs. fraction of O_2 uncoupling, $f_{O_2\text{-uc}}$. The top panel shows data for WT and variants of the $4NT^+$ experiment (M248I, L238V-M248I). The bottom panel displays the corresponding data for WT and variants of the $3NT^+$ experiment (I204A, I204T, I204V). The solid lines are linear regressions.

S3.4 Enzyme Kinetics

Kinetic parameters for the dioxygenation of nitroaromatic substrate, approximated by NO_2^- formation, and O_2 activation by 2NTDO variants are shown in Table S12.

Table S12 Kinetic parameters for the dioxygenation of nitroaromatic substrate (S) dioxygenation and O_2 activation by 2NTDO variants.

Substrate	species	v_{max} (μM s^{-1})	k_{cat} (s^{-1})	K_m (μM)	k_{cat}/K_m (10^3 M^{-1} s^{-1})	v_0[b] (μM min^{-1})
M248I						
nitrobenzene	(O_2)	1.31 ± 0.07	2.91 ± 0.16	11.3 ± 3.8	257.3 ± 105.1	73.2 ± 6.3
2-nitrotoluene	(S)	0.37 ± 0.05	0.82 ± 0.11	37.4 ± 14.2	21.9 ± 13.7	n.a.[c]
	(O_2)	0.83 ± 0.02	1.84 ± 0.04	14.7 ± 1.5	126.0 ± 15.4	36.7 ± 0.5
3-nitrotoluene	(O_2)	1.07 ± 0.07	2.38 ± 0.16	13.9 ± 4.8	172.2 ± 74.5	53.0 ± 7.1
4-nitrotoluene	(S)	0.23 ± 0.03	0.51 ± 0.07	65.6 ± 23.1	7.8 ± 4.0	n.a.
	(O_2)	1.13 ± 0.04	2.51 ± 0.09	17.5 ± 2.8	143.4 ± 27.8	49.7 ± 1.7
L238V-M248I						
nitrobenzene	(O_2)	0.96 ± 0.06	2.13 ± 0.13	10.0 ± 3.9	212.5 ± 102.6	43.5 ± 4.7
2-nitrotoluene	(S)	0.66 ± 0.14	1.47 ± 0.31	54.6 ± 27.7	26.8 ± 20.9	n.a.
	(O_2)	0.82 ± 0.04	1.82 ± 0.09	19.2 ± 4.3	95.1 ± 25.9	33.1 ± 1.0
3-nitrotoluene	(O_2)	0.75 ± 0.01	1.67 ± 0.02	17.3 ± 1.2	96.2 ± 8.4	36.9 ± 0.6
4-nitrotoluene	(S)	0.43 ± 0.03	0.96 ± 0.07	110 ± 20	8.6 ± 2.3	n.a.
	(O_2)	1.36 ± 0.04	3.02 ± 0.09	16.9 ± 2.6	178.2 ± 32.9	61.6 ± 3.4
I204T						
nitrobenzene	(O_2)	0.32 ± 0.01	0.72 ± 0.02	33.2 ± 2.9	21.6 ± 2.5	14.0 ± 0.2
2-nitrotoluene	(O_2)	0.25 ± 0.01	0.56 ± 0.02	74.1 ± 5.2	7.6 ± 0.8	7.8 ± 0.1
3-nitrotoluene	(O_2)	0.28 ± 0.02	0.63 ± 0.04	27.3 ± 6.9	23.1 ± 7.3	12.5 ± 0.7
4-nitrotoluene	(O_2)	0.18 ± 0.01	0.40 ± 0.03	109.2 ± 15.0	3.6 ± 0.8	6.9 ± 0.1
I204A						
nitrobenzene	(O_2)	0.35 ± 0.01	0.77 ± 0.04	30.2 ± 4.7	25.4 ± 5.3	13.0 ± 1.2
2-nitrotoluene	(O_2)	0.28 ± 0.01	0.62 ± 0.02	50.7 ± 4.1	12.1 ± 1.4	9.3 ± 0.1
3-nitrotoluene	(O_2)	0.40 ± 0.01	0.89 ± 0.02	29.8 ± 2.4	29.9 ± 3.1	16.3 ± 0.2
4-nitrotoluene	(O_2)	0.09 ± 0.01	0.20 ± 0.01	37.0 ± 5.5	5.4 ± 1.0	4.3 ± 0.1
I204V						
nitrobenzene	(O_2)	0.45 ± 0.03	1.01 ± 0.07	27.8 ± 7.4	36.3 ± 12.2	22.9 ± 0.9
2-nitrotoluene	(O_2)	0.44 ± 0.01	0.98 ± 0.02	27.3 ± 2.1	35.8 ± 3.5	23.0 ± 0.2
3-nitrotoluene	(O_2)	0.27 ± 0.01	0.60 ± 0.02	25.3 ± 3.34	23.8 ± 3.9	7.8 ± 0.2
4-nitrotoluene	(O_2)	0.22 ± 0.01	0.48 ± 0.02	39.2 ± 4.3	12.3 ± 1.9	11.5 ± 0.5

[a] Kinetics of nitroaromatic substrate (S) dioxygenation were quantified based on NO_2^- formation and equation S1; kinetics of O_2 activation were were based on equation S2; uncertainties correspond to 95% confidence intervals; [b] initial rates of O_2 consumption were determined for the first minute after reaction initiation through NADH-addition; [c] n.a. = not applicable.

S3.5 Qualitative comparison of fitness parameters of *Acidovorax* sp. strain JS42 and evolved 4NT$^+$ and 3NT$^+$ strains

Table S13 Overview of fitness parameters of 2NTDO and variants adapted to 4-nitrotoluene or 3-nitrotoluene. "G" and "nG" stand for "growth" and "no growth", respectively, of *Acidovorax* sp. strain JS42 expressing the respective enzymes. "+" and "–" are an increase or decrease in O_2 uncoupling, $\Delta f_{O_2\text{-uc}}$, of less than 10%, respectively. "++" and "–" indicate changes of more than 10%. Data on growth were taken from Ju and Parales[11] and Mahan et al.[3].

Experiment		Substrates							
type	enzyme	NB		2-NT		3-NT		4-NT	
		growth	$\Delta f_{O_2\text{-uc}}$	growth	$\Delta f_{O_2\text{-uc}}$	growth	$\Delta f_{O_2\text{-uc}}$	growth	$\Delta f_{O_2\text{-uc}}$
wt	2NTDO	G	0	G	0	–	0	–	0
4NT$^+$	M248I	n.d.[a]	+	G	+	–	–	G	– –
	L238V-M248I	n.d.	+	G	+	–	–	G	– –
3NT$^+$	I204T	nG	+ +	G/nG[b]	+ +	G	– –	n.d.	–
	I204V	G	+ +	G	+ +	G	– –	n.d.	n.d.
	I204A	nG	+ +	nG	+ +	G	– –	n.d.	n.d.

[a] n.d. = no data; [b] expression in the evolved strains supported growth, but not in unexposed strains complemented with the mutant *ntdAcAd* genes.

S3.6 Significance of oxidative stress-induced oxidation of nucleic acids

G→T transversions arising from the oxidation of guanine to 8-oxoguanine represent the most likely mutations induced by oxidative stress.[12,13] We tested their relevance for mutations that could lead to the expression of enzyme variants with improved O_2 uncoupling behavior.

The codons for the I204, L238, and M248 residues are shown in the nucleotide sequence of the *ntdAc* gene for the oxygenase α-subunit of wt 2NTDO (Figure S10). Enzyme variant M248I could have arisen from G→T transversion of codons ATG (Met) to ATT (Ile) lending support for the overarching hypothesis that ROS-induced pressure from O_2 uncoupling ultimately resulted in variant M248I. No such connections can be made for variant I204 (codon ATC). G→T mutation of TTG at position L238 to TTT would have resulted in L238F instead of the observed

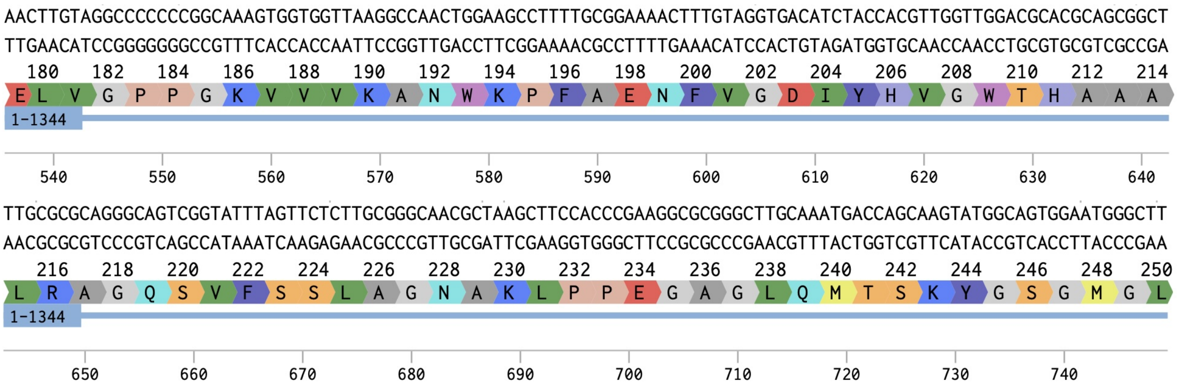

Figure S10 Nucleotide sequence of the *ntdAc* gene for the oxygenase α-subunit of wt 2NTDO aligned with the corresponding amino acid residues. NIH/NCBI Accession U49504.1:2523-3866 (accessed April 17, 2024).

L238V. We note, however, that other mutations and their possible relationship to oxidative stress were not considered here.

S3.7 Additional experiments for the comparison of *in vivo* evidence for adaptation with oxygenation efficiencies quantified *in vitro*

Figure S11 shows data analogous to Figure 7 (a) to (c) in the main manuscript but data for $3NT^{+}$ experiments (panel a) as well as for reconstituted JS42 strains (panels b, c) instead of evolved ones. All y-axis data were obtained from the works of Ju and Parales[11] and Mahan et al.[3]

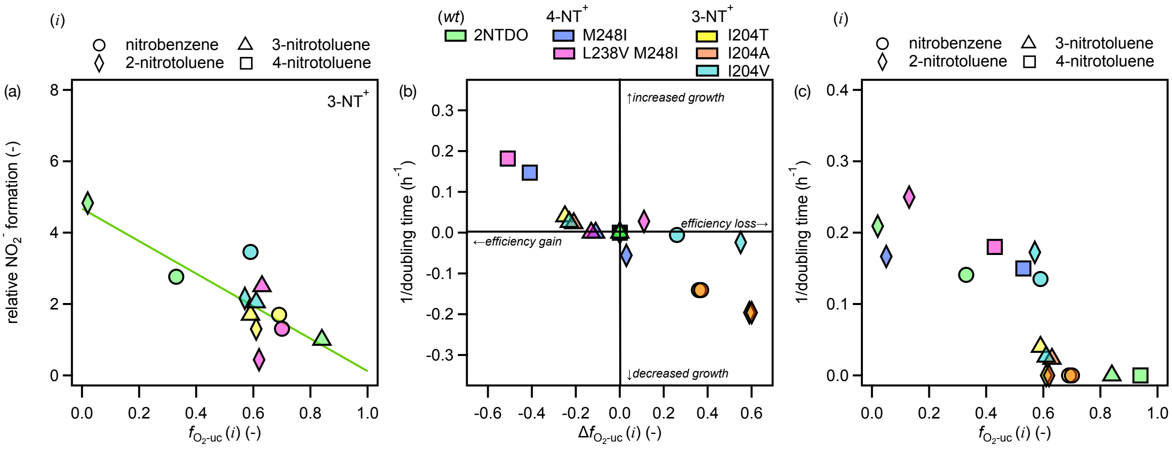

Figure S11 (a) Rates of nitrate formation by *Acidovorax* sp. J42 and evolved $3NT^{+}$ strains (per mg of protein) normalized to value obtained for 3-nitrotoluene by wt 2NTDO vs. $f_{O_2\text{-uc}}$ from enzyme assays with wt 2NTDO and variants from $3NT^{+}$ experiments for 4 substrates. (b) Changes of inverse doubling times, Δ(1/doubling time), of *complemented* strains of *Acidovorax* sp. JS42 relative to inverse doubling times determined with *Acidovorax* sp. J42 strain and the 4 substrates vs. changes of O_2 uncoupling, $\Delta f_{O_2\text{-uc}}$, of variants relative to $f_{O_2\text{-uc}}$ obtained with the 4 substrates with wt 2NTDO. (c) Inverse doubling times vs. $f_{O_2\text{-uc}}$ without normalization. Note that all data used for y-axes originate from whole cell experiments and were obtained from Ju and Parales[11] and Mahan et al.[3]

S3.8 Natural occurrence of 2NTDO homologs

Analysis of sequencing data confirmed the presence of the M248I, L238V-M248I, I204T, I204A, and I204V variants in naturally-occurring environmental sequences. Up to 25% of the 2NTDO homologs queried encode one or more of the five mutations of interest, indicating a relatively high natural abundance and taxonomic distribution (Figure S12). Consistently across different genomic and metagenomic datasets, I204A was the most commonly detected protein variant detected in 7 - 17% of dioxygenase sequences examined. Relative abundances of each protein variant differ depending on the dataset and are heavily influenced by the bias of sequencing databases. However, all protein variants of interest were detected in all major types of environments (e.g., marine, freshwater, terrestrial) sampled. Overall, these results indicate that the RDO variants examined in this study are not only products of laboratory evolution but also have arisen through natural evolutionary processes in the environment.

We also analyzed the co-occurrence of M248I-L238V variants hypothesized by Parales and co-workers[3,11] to be a sequential process, and also observed this double variant to occur in

nature, albeit rare. However, the individual M248I and L238V mutations are far more common, and are abundant "precursors" for the L238V-M248I doubly-substituted variant with higher 4-nitrotoluene oxidation activity to arise under appropriate selective pressure (e.g., a 4-nitrotoluene polluted environment), despite the added cost of higher O_2 uncoupling.

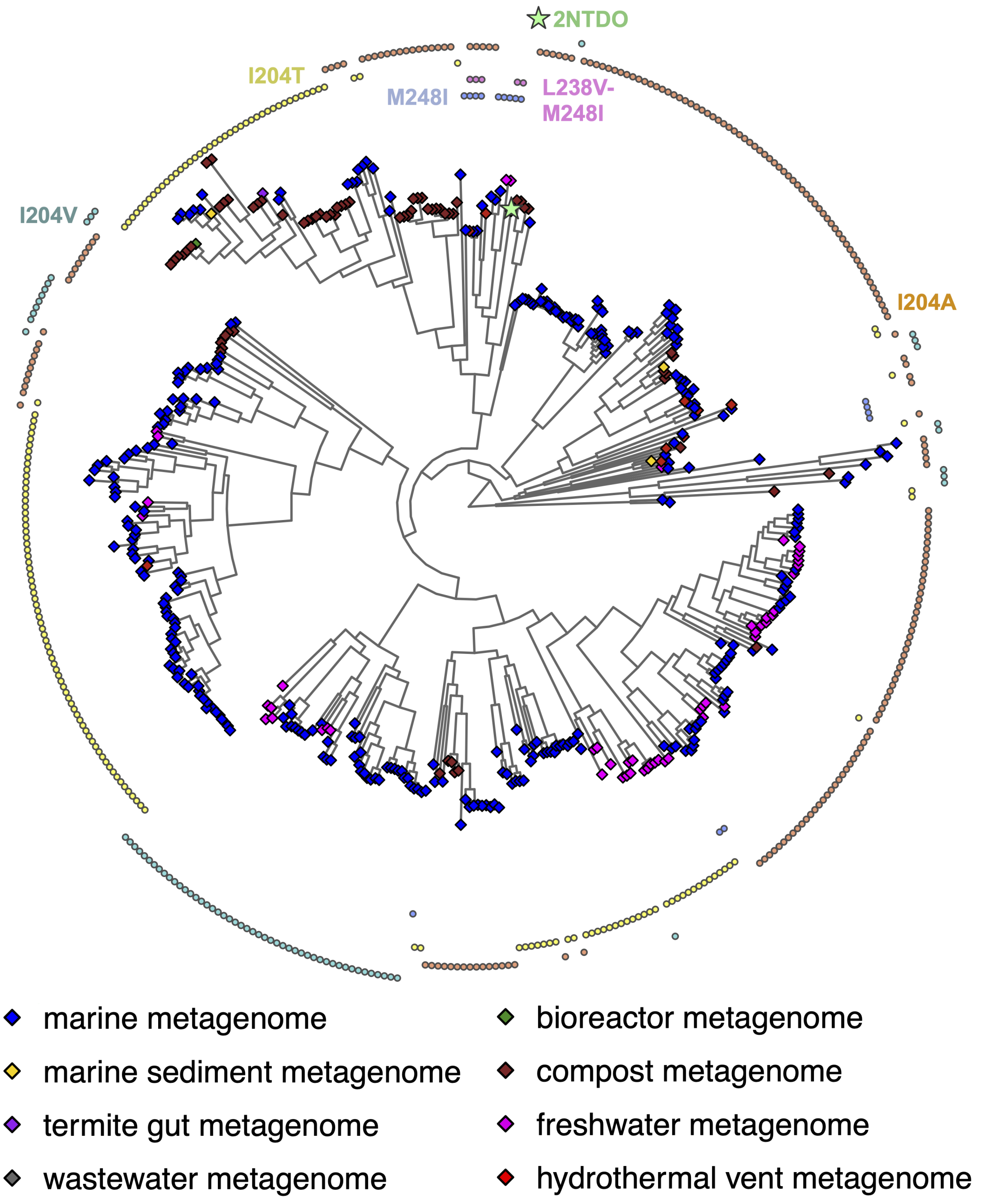

Figure S12 Homologs for *Acidovorax* sp. strain JS42 2NTDO (AAB40383.1) in WGS metagenomic projects. Markers in the circle indicate the mutation detected. Colors at the tree endpoints refer to the metagenome environment.

References

[1] Bopp, C. E.; Bernet, N. M.; Kohler, H.-P. E.; Hofstetter, T. B. Elucidating the role of O_2 uncoupling in the oxidative biodegradation of organic contaminants by Rieske non-heme iron dioxygenases. *ACS Environmental Au* **2022**, *2*, 428–440, https://doi.org/10.1021/acsenvironau.2c00023.

[2] Parales, R. E.; Huang, R.; Yu, C. L.; Parales, J. V.; Lee, F. K. N.; Lessner, D. J.; Ivkovic-Jensen, M. M.; Liu, W.; Friemann, R.; Ramaswamy, S.; Gibson, D. T. Purification, characterization, and crystallization of the components of the nitrobenzene and 2-nitrotoluene dioxygease enzyme systems. *Appl. Environ. Microbiol.* **2005**, *71*, 3806–3814, https://doi.org/10.1128/AEM.71.7.3806-3814.2005.

[3] Mahan, K. M.; Penrod, J. T.; Ju, K.-S.; Al Kass, N.; Tan, W. A.; Truong, R.; Parales, J. V.; Parales, R. E. Selection for growth on 3-nitrotoluene by 2-nitrotoluene-utilizing *Acidovorax* sp. strain JS42 identifies nitroarene dioxygenases with altered specificities. *Appl. Environ. Microbiol.* **2015**, *81*, 309–319, https://doi.org/10.1128/aem.02772-14.

[4] Madeira, F.; Pearce, M.; Tivey, A. R. N.; Basutkar, P.; Lee, J.; Edbali, O.; Madhusoodanan, N.; Kolesnikov, A.; Lopez, R. Search and sequence analysis tools services from EMBL-EBI in 2022. *Nucleic Acids Research* **2022**, *50*, W276–W279, https://doi.org/10.1093/nar/gkac240.

[5] Robert, X.; Gouet, P. Deciphering key features in protein structures with the new ENDscript server. *Nucleic Acids Research* **2014**, *42*, W320–W324, https://doi.org/10.1093/nar/gku316.

[6] An, D.; Gibson, D. T.; Spain, J. C. Oxidative release of nitrite from 2-nitrotoluene by a three-component enzyme system from *Pseudomonas* sp. strain JS42. *J. Bacteriol.* **1994**, *176*, 7462–7467, https://doi.org/10.1128/jb.176.24.7462-7467.1994.

[7] Pati, S. G.; Kohler, H.-P. E.; Pabis, A.; Paneth, P.; Parales, R. E.; Hofstetter, T. B. Substrate and enzyme specificity of the kinetic isotope effects associated with the dioxygenation of nitroaromatic contaminants. *Environ. Sci. Technol.* **2016**, *50*, 6708–6716, https://doi.org/10.1021/acs.est.5b05084.

[8] Pati, S. G.; Bopp, C. E.; Kohler, H.-P. E.; Hofstetter, T. B. Substrate-specific coupling of O_2 activation to hydroxylations of aromatic compounds by Rieske non-heme iron dioxygenases. *ACS Catal.* **2022**, *12*, 6444–6456, https://doi.org/10.1021/acscatal.2c00383.

[9] Bopp, C. E.; Kohler, H.-P. E.; Hofstetter, T. B. Enzyme kinetics of organic contaminant oxygenations. *Chimia* **2020**, *74*, 108–114, https://doi.org/10.2533/chimia.2020.108.

[10] Strathmann, T. J. In *Aquatic Redox Chemistry*; Tratnyek, P. G., Grundl, T. J., Haderlein, S. B., Eds.; ACS Symposium Series; American Chemical Society, 2011; Vol. 1071; pp 283–313, https://doi.org/10.1021/bk-2011-1071.ch014.

[11] Ju, K. S.; Parales, R. E. Evolution of a new bacterial pathway for 4-nitrotoluene degradation. *Mol. Microbiol.* **2011**, *82*, 355–364, https://doi.org/10.1111/j.1365-2958.2011.07817.x.

[12] Sies, H.; Berndt, C.; Jones, D. P. Oxidative stress. *Annu. Rev. Biochem.* **2017**, *86*, 715–748, https://doi.org/10.1146/annurev-biochem-061516-045037.

[13] Hahm, J. Y.; Park, J.; Jang, E.-S.; Chi, S. W. 8-Oxoguanine: From oxidative damage to epigenetic and epitranscriptional modification. *Exp Mol Med* **2022**, *54*, 1626–1642, https://doi.org/10.1038/s12276-022-00822-z.
